# Interpreting Neural Network Models for Toxicity Prediction by Extracting Learned Chemical Features

**DOI:** 10.1021/acs.jcim.4c00127

**Published:** 2024-04-30

**Authors:** Moritz Walter, Samuel J. Webb, Valerie J. Gillet

**Affiliations:** † Information School, University of Sheffield, The Wave, 2 Whitham Road, Sheffield S10 2AH, U.K.; ‡ Lhasa Limited, Granary Wharf House, 2 Canal Wharf, Leeds LS11 5PY, U.K.

## Abstract

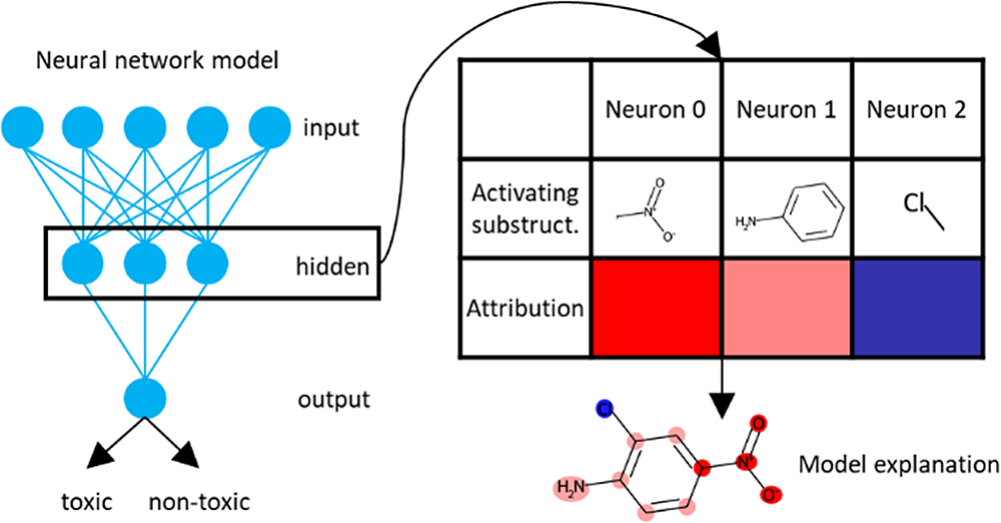

Neural network models have become a popular machine-learning technique for the toxicity prediction of chemicals. However, due to their complex structure, it is difficult to understand predictions made by these models which limits confidence. Current techniques to tackle this problem such as SHAP or integrated gradients provide insights by attributing importance to the input features of individual compounds. While these methods have produced promising results in some cases, they do not shed light on how representations of compounds are transformed in hidden layers, which constitute how neural networks learn. We present a novel technique to interpret neural networks which identifies chemical substructures in training data found to be responsible for the activation of hidden neurons. For individual test compounds, the importance of hidden neurons is determined, and the associated substructures are leveraged to explain the model prediction. Using structural alerts for mutagenicity from the Derek Nexus expert system as ground truth, we demonstrate the validity of the approach and show that model explanations are competitive with and complementary to explanations obtained from an established feature attribution method.

## Introduction

Quantitative structure–activity relationship (QSAR) models are statistical models that attempt to link the chemical structure of a compound to a measured bioactivity. QSAR modeling has seen extensive applications in drug discovery and toxicity assessment^1^ with many different chemical descriptors and machine learning (ML) techniques used. Typical machine learning methods include k-nearest neighbors,^2^ support vector machines,^3^ random forest,^4^ gradient tree boosting,^5^ and more recently deep neural networks (DNN).^6^ Chemical fingerprints are widely used as input features, however, a recent focus has been on the use of different types of descriptors including, for example, string representations such as SMILES,^7,8^ depictions of chemical structures as inputs to DNNs,^9^ and 2D and 3D chemical graphs^10−12^ which have been used with both classical machine learning methods and with more novel graph-based DNN architectures.

DNNs have gained a lot of attention for QSAR modeling following successes in modeling competitions.^6,13^ When traditional chemical descriptors are selected as input, feedforward neural network architectures are used. These consist of one input layer, one or more hidden layers (when there is more than one hidden layer neural networks (NNs) are referred to as deep),^14^ and one output layer. The activation of neurons in the hidden layers for a given input consists of a linear combination of the neurons in the previous layer, followed by a nonlinear transformation. This gives NNs the flexibility to fit complex relationships between the input and the modeled output. While DNNs may be able to generate more accurate models than classical machine learning methods, they are often referred to as “black box” methods due to their apparent lack of interpretability^15^ and, there may be a trade-off between interpretability and performance for more complex problems.

Having the ability to interpret the predictions made by a model can increase its utility both in determining what molecule to make next (e.g., in a drug discovery project) as well as in a regulatory context. For example, in the case of toxicity prediction, when a model predicts the presence of a hazard, understanding the cause for the prediction in terms of the presence of a particular structural motif can provide insight on where to focus modifications to the structure to mitigate the risk. In the regulatory context and in the case of mutagenicity prediction, the ICH (International Council for Harmonisation of Technical Requirements for Pharmaceuticals for Human Use) M7 guidelines on the assessment and control of DNA reactivity impurities allow for the use of negative predictions from two complementary QSAR systems to reason that the impurity is of no mutagenic concern.^16^ These systems should adhere to the Organisation for Economic Co-operation and Development (OECD) guidelines for (Q)SAR modeling where principle 5 states that predictions should be associated with “a mechanistic interpretation, if possible”.^17^ Furthermore, expert review may be required such as when the systems disagree. In such cases, the more information a system can provide the easier it is to reason about the prediction, and therefore interpretation can become an important facet to use when performing the expert review.

Recently, several approaches to achieve interpretability for NNs in the context of QSAR modeling have been described.^18^ Most widely used are techniques that assign importance to the input features of the model. This can be done either globally (i.e., the importance of features for the model’s overall performance) or locally (i.e., the importance of features for individual predictions made by the model).^19^ Methods that determine local feature importance are also called attribution methods. Some attribution methods have been specifically developed for NNs such as integrated gradients (IG),^20^ while others can be applied to any ML technique (e.g., Local Interpretable Model-Agnostic Explanations (LIME),^21^ SHapley Additive exPlanations (SHAP),^22^ and perturbation methods^23,24^).

Some studies have attempted to interpret the chemical information that is learned in the hidden layers of a NN. In analogy to classical chemical fingerprints, the activations of neurons in a hidden layer can be considered as a neural fingerprint that has learned features of a training set.^25^ In the cited work, the neural fingerprint was used in a similarity-based virtual screening experiment. A DNN was first trained to predict activity for the target of interest and then a query compound was input to the model and its neural fingerprint used to identify compounds with similar neural fingerprints. In another study, Sosnin et al. used DNNs to predict acute toxicity and analyzed the hidden layer representations of chemicals with the t-SNE (t-distributed stochastic neighbor embedding) method, which embeds them in a 2D space.^26^ Distinct clusters of compounds having high acute toxicity emerged, which presumably correspond to different mechanisms of toxicity. These studies demonstrate that hidden representations of chemicals in NNs are meaningful in the context of the investigated bioactivity or toxicity tasks, although the meaning of those hidden representations is not well understood.

Unpacking the information learned by NNs has been studied extensively in image recognition tasks. For example, it has been shown that a convolutional neural network (CNN) trained on images constructs features of increasing complexity throughout the different layers of a network.^27^ Thus, when detecting faces lower layers detect simple structures like blobs and edges from the raw pixels, while deeper layers combine the simple structures into more complex objects such as eyes and noses. Different techniques, referred to as feature visualization or activation maximization, have been developed to understand what visual patterns are detected by individual hidden neurons.^28,29^ These techniques include: inspecting exemplary images that strongly activate a neuron;^30^ optimizing images in the input space to strongly activate a neuron;^31^ and using generative models to create images that strongly activate a neuron.^32^

Analogously, when learning representations for chemicals, it could be that a NN detects the presence of simple substructures in the lower layers and combines these with more complex substructures that are meaningful for the task at hand. Some attempts have been made to understand the chemical features learned in hidden neurons of a NN. For example, it was shown that the activation of hidden neurons can be correlated with the presence of toxicophores (known toxicophores for various toxicity end points were considered) in the compounds of the Tox21 data set.^13^ Furthermore, it was shown that the size of the detected toxicophores (in number of atoms) increases in deeper layers.^33^ However, these studies did not investigate if the detected toxicophores are related to the modeled toxicity end points of the Tox21 data set. In principle, a hidden neuron may be responsive to a chemical pattern without the network using this information for the eventual prediction. The two cited studies shed some light on the mechanisms by which NNs may learn features, but no attempts were made to leverage this information to interpret predictions made by a specific model.

Attribution methods that operate on the input data can only study the impact of individual features on a prediction independently of each other. In contrast, the activation of hidden neurons corresponds to a nonlinear transformation of input features learned explicitly to solve the prediction task at hand. Hence the hidden representations can be associated with chemical substructures that are distinct from those identified by analyzing information provided in the input layer.

In this study, we describe the development and validation of a method to interpret predictions made by DNNs by extracting information encoded in hidden layers of a NN. Our approach is aimed at providing explanations for end points where the activity is due to the presence of particular chemical substructures, hence the use of structural fingerprints as descriptors. We have validated the approach on the Ames mutagenicity data set since this is a well-understood data set where the causes of toxicity are known and can therefore provide ground truth. While there are other data sets that are relevant for toxicity prediction such as Tox21, previous studies on this data have mainly focused on prediction performance and the data are not so well understood in terms of reasoning about toxicity. We first demonstrate that the activation of hidden neurons is linked to the presence of toxicophores for mutagenicity. Next, we describe a method to automatically identify chemical substructures found to activate a given hidden neuron. Finally, we use these substructures to interpret individual predictions made by a NN. The model explanations are evaluated by comparing to the ground truth (i.e., substructures that are known to be linked to mutagenicity) and by comparison with an established IG approach based on assigning importance to input features.

## Methodology

### Data Sets

Ames mutagenicity was selected as the studied toxicity end point as it represents a well-understood mechanism of toxicity with many different known toxicophores.^34,35^ This means that structural features identified by a NN can be compared to known toxicophores as a form of validation. Hence the data set allows us to systematically evaluate the quality of model explanations provided by different model interpretation techniques. The Ames data set used here was constructed by combining data from the following public sources: a curated version of the Hansen data set,^36,37^ the ISSSTY data set,^38^ the EURL-ECVAM Ames positive DB,^39^ the CGX database^40^ and the Genotoxicity and Carcinogenicity database for marketed pharmaceuticals.^41^ The ISSSTY data set contains data on compounds that have been tested against a number of different bacteria strains with each compound also labeled with the “overall call” (positive if at least one strain is positive). The overall call was used here and only compounds labeled as negative or positive were kept; compounds labeled as equivocal or inconclusive were removed. The other sources contain compounds with an overall call only (that is, a single label). The compounds in the curated Hansen data set have binary labels and no further changes were made. For the EURL-ECVAM data set, compounds labeled as equivocal were removed. This data set did not contain SMILES strings and so, where possible, SMILES strings were retrieved using CAS numbers and the CIRpy package in Python (Version 1.0.2).^42^ For the remaining data sources, compounds with missing or equivocal labels were removed. After these processing steps, each compound was labeled with a binary outcome for the Ames Test.

Subsequently, the SMILES of all compounds were standardized using RDKit (Version 2021.03.3)^43^ and MolVS (Version 0.1.1).^44^ In particular, metal atoms, inorganic fragments, and solvents were removed and, where possible, charges were neutralized. Chemotypes and tautomers were transformed into a canonical form. Duplicates were identified by calculating InChIs^45^ with the InChIs being converted back to SMILES. Compounds consisting of mixtures of different organic components were discarded. Finally, data instances with identical SMILES were aggregated, and the majority vote of the labels was used. If equal numbers of positive and negative labels were found for a given SMILES, the compound was removed from the final data set. The final data set consists of 7662 compounds.

In addition to using experimental Ames labels, the Derek Nexus software^46^ was used to label the compounds according to the presence of structural alerts for mutagenicity. This was done to obtain a labeling that is defined by clear rules and is not subject to experimental uncertainty. Of the 7662 unique original structures, 7336 could be processed in Derek Nexus. The remaining structures were discarded. The Derek Nexus software returned an SDF (structure-data file) containing each compound structure in a MolFile (connection table) format, the alerts matched by the compound (with more than one possible), and the atoms of the substructure responsible for the alert(s) being matched. According to its internal rules, the Derek Nexus software labels compounds as “INACTIVE”, “EQUIVOCAL”, “PLAUSIBLE” or “PROBABLE”. The latter three categories indicate the presence of one or more alerts and those compounds were labeled as the “positive” (i.e., toxic) class, with the others labeled as “negative” (i.e., nontoxic). Across the whole data set, 105 distinct alerts were fired and each of these was assigned an identifier (Alert1–Alert105). Note that these identifiers are distinct from alert identifiers in the Derek Nexus software and are used here for reference in the text.

### Model Training

Three feedforward NN models were trained: a single-layer model trained on experimental Ames labels; a single-layer model trained on Derek labels; and a two-layer model trained on Derek labels. All models were trained using RDKit’s Morgan fingerprints (FP) with radius 1, hashed to 2048 bits as input features. In all cases, the NN was trained on 80% of the data (random split), with 10% used as a validation set for early stopping of model training and a further 10% retained as a test set for final model evaluation. The same splits were used for all the models. Model hyperparameters that differed between the models are reported in Table 1. In all cases, the hidden layer(s) of the models contained 512 neurons. The ReLU activation function was used, and the models were trained for a maximum of 10 epochs using batches of size 16 and the Adam optimizer. Early stopping was employed to prevent overfitting. Specifically, the performance on the validation set was recorded after each epoch, and the best-performing model (ROC-AUC score) instance (after a particular epoch) was retained. The loss was evaluated using binary cross entropy. The models were implemented in Pytorch (Version 1.9.0),^47^ and the model instance trained on experimental Ames data (one-layer Ames) is shared in the accompanying code repository.

**Table 1:** Neural Network Hyperparameters

hyperparameter	one-layer Ames	one-layer Derek	two-layer Derek
number of hidden layers	1	1	2
learning rate	0.001	0.001	0.0001
dropout in hidden layer	0.5	0.5	0.2
L2 regularization of hidden neuron weights	0.001	0.001	0

### Overview of Methods

The first step of the method is to associate chemical substructures with each of the neurons in a trained NN. Chemical substructures are identified for each neuron by combining information about input features (bits of the Morgan FP) that have high learned weights with training compounds that strongly activate the neuron. The rationale is to first identify combinations of input features and then to identify substructures consistent with these by examining the training compounds that activate the neuron strongly. Thus, the atom environments corresponding to the highly weighted bits are searched in the strongly activating training compounds. However, not all the bits will be present in all the compounds and formal concept analysis (FCA), described below, is used to identify subsets of bits and compounds from which substructures are identified. This process is applied to each hidden neuron in the NN so that each neuron is represented by a set of substructures that are associated with activation. The substructures can then be used to highlight features of a test compound that give rise to the prediction made using the following procedure. First, the importance of each neuron in making a prediction is determined. Then, for each neuron, its associated substructures are matched to the test compound, and the corresponding atoms of the compound are weighted according to the importance of each neuron in making the prediction. The weights (or atom attributions) are aggregated over all neurons to take account of all neurons in the network and to provide an overall explanation for the prediction.

Each of the steps is described in detail below. The methodology contains several hyperparameters that can be selected by the user. These are presented below (and written in italics) along with the hyperparameter values used for the reported experiments.

### Substructure Extraction Method

**Figure 1:**
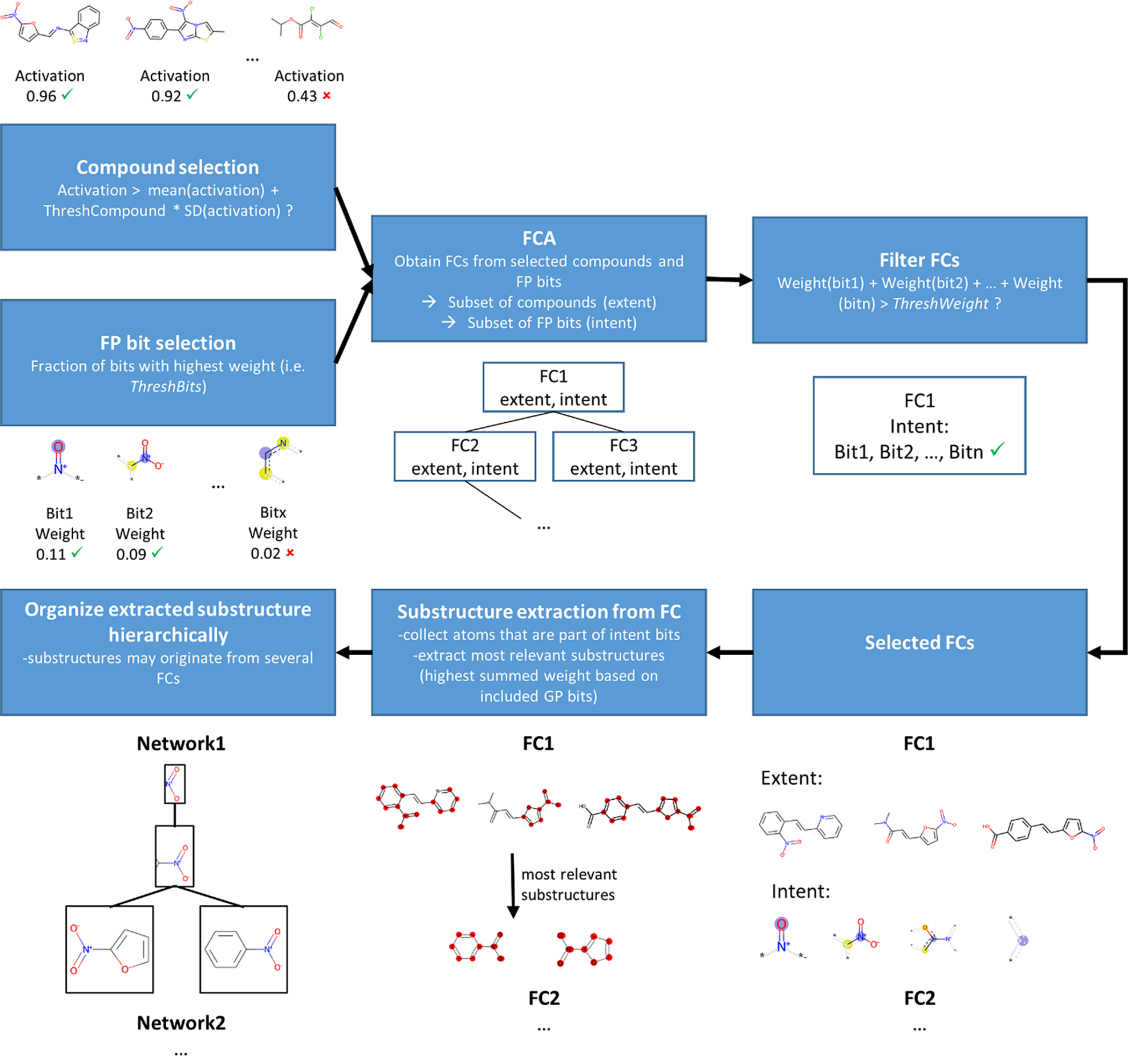
Overview of the substructure extraction method. For individual hidden neurons FCA is applied to compounds strongly activating the neuron and FP bits with high weights. Substructures are extracted from relevant FCs and organized hierarchically. Detailed explanations can be found in the main text.

The method for identifying substructures associated with a neuron is illustrated in Figure 1. The subset of training compounds that are identified as strongly activating a neuron is determined using a threshold on the compound’s activation value. The threshold applied is that the activation value should be greater than two standard deviations from the mean value of the activation values for the neuron (*ThreshCompound* = 2). Similarly, the subset of input features (or bits in the Morgan FP) is determined using a threshold on the learned weights for the neuron. The threshold used is that the learned weight of a feature should be at, or above, the 90th percentile considering the magnitudes of all the weights connecting the input feature to the neuron (*ThreshBits* = 0.1). This means that the 10% of bits with the highest learned weights are chosen. The selected training compounds and input features are then subjected to FCA as described below. The distributions of activation values and learned weights are shown below in the Results section for an example neuron (see Figure 5).

**Figure 2:**
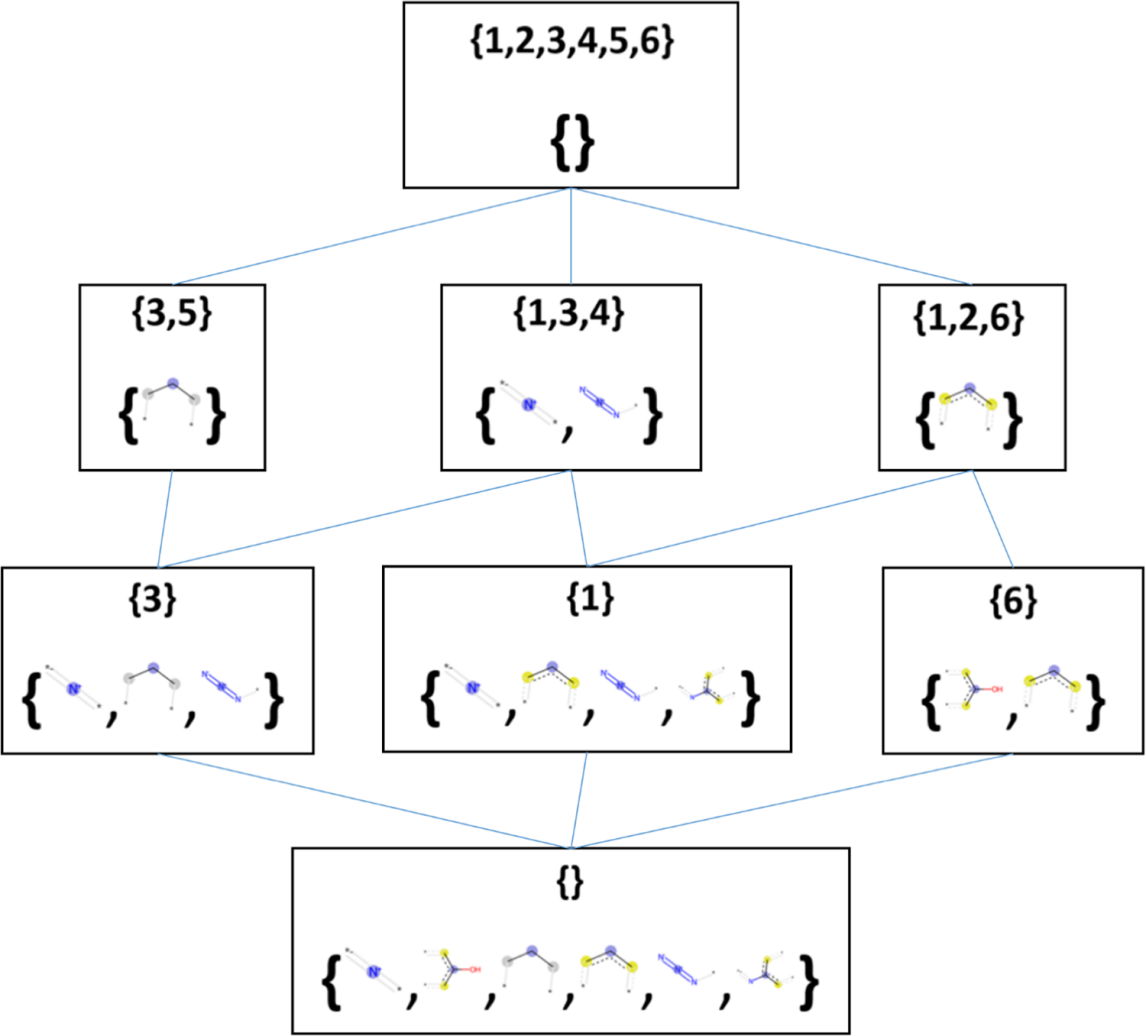
Hasse diagram depicting the lattice derived using FCA. Each box contains a FC consisting of an extent (set of compound identifiers in the first line) and an intent (set of FP bits). None of the considered bits is shared by all the compounds (hence the empty intent in the FC at the top) and none of the compounds sets all the FP bits on (hence the empty extent in the FC at the bottom). The remaining FCs describe a certain chemical concept defined by a set of FP bits and all compounds having those bits set on.

FCA was introduced as a method for hierarchically organizing data into “formal concepts”^48^ and has been used previously in chemoinformatics to mine substructures associated with bioactivity/toxicity.^49^ A formal concept (FC) in FCA is a triple (U, A, R) consisting of sets of objects U (i.e., the extent), sets of attributes A (i.e., the intent), and binary relations R (indicating whether an object u possesses attribute a). Here, objects are chemical compounds, and their attributes are bits of the Morgan FP indicating the presence of certain atom environments in the compounds. The binary relations describe whether a compound has a given FP bit set on. In a FC, all objects represented by the FC share all the attributes of the FC. Furthermore, the FC is closed in the sense that there are no further attributes shared by all the objects, and, in turn, no further objects exist that possess all included attributes. A hierarchical lattice (i.e., a Hasse diagram) consisting of all existing FCs for a given data set can be derived. An illustrative example of how FCA is applied here is shown in Figure 2 and Table 2. Table 2 shows a set of compounds and a set of FP bits and indicates which bits are set to “on” in which compounds. Figure 2 shows how the compounds and FP bits are arranged hierarchically as a Hasse diagram. Each box corresponds to a FC and consists of a set of compounds and a set of FP bits. The FC at the top of the Hasse lattice represents all the identified bits and the subset of compounds that contain them all (which may be none as is shown in Figure 2). As the Hasse lattice is descended, the FCs contain fewer bits but more compounds. For instance, the FC with compounds 1, 3, and 4 in the extent contains all the azides (which are characterized by FP bits 487 and 1838), whereas the FC with only compound 1 in the extent contains only aromatic azides (characterized by FP bits 487, 1838, and 1854) and hence is more specific. The latter FC (aromatic azides) is a subconcept of the former (generic azides) and vice versa, i.e., the former is a superconcept of the latter. A FC, therefore, corresponds to a chemical concept defined by a set of atom environments and the training compounds that contain these environments (i.e., for which all the bits in the FC are set “on”). In this work, we used the implementation of FCA in the Python package concepts (Version 0.9.2).^50^

**Table 2:**
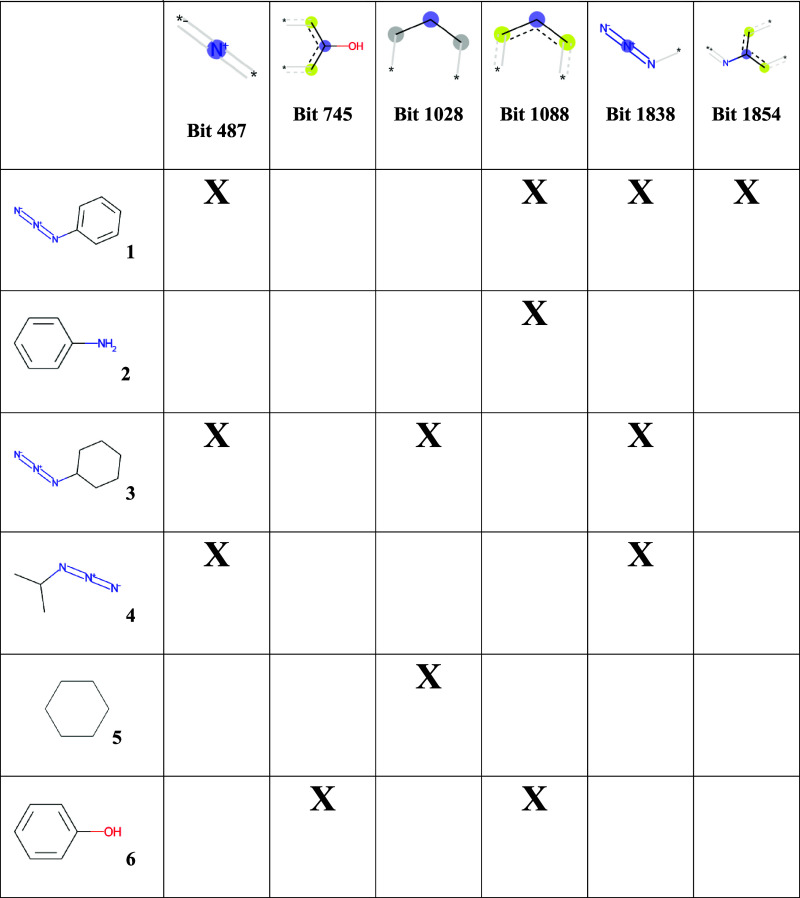
Binary Relations between Compounds and FP Bits As Basis for FCA^a^

a A small number of compounds and FP bits was selected to illustrate the foundations of FCA. The table indicates which FP bits are set on for each compound.

The next stage of the substructure extraction process is to convert each FC into a set of chemical substructures. For each compound in the extent of a FC, atoms matching any of the FP bits contained in the intent are identified using the atom environments provided in the RDKit. Then, connected substructures are obtained by connecting neighboring identified atoms. Since not all identified atoms are necessarily connected, more than one connected substructure may be obtained for each compound.

The substructure extraction process is controlled by several parameters which aim to ensure that the substructures are meaningful. First, not all FCs will correspond to chemical substructures of interest. For instance, a FC may contain just a single FP bit and this feature alone may not be sufficient for strong neuron activation. Hence, only FCs whose intent (set of FP bits) reaches a certain relevance were considered. This is assessed by calculating the sum of weights of the fingerprint bits in the intent and applying a threshold. The threshold (*ThreshWeight*) is defined as a fraction of the threshold described above for the inclusion of compounds (*ThreshCompounds*). In the results presented here, *ThreshWeight* was selected to be 1 (i.e., the same value as for *ThreshCompounds*) as lower fractions were not found to be beneficial (results not shown).

Second, only the most relevant substructure for each compound is retained. This is the substructure with the highest sum of weights for bits included in the substructure (which may be a subset of the bits of the intent). Third, only substructures causing a sufficiently strong activation on the neuron are included. Thus, a substructure is only retained if the sum of weights is higher than a given threshold. Here the same threshold was used as applied to the summed weights of all bits of the intent (i.e., *ThreshWeight*, see above). Finally, a substructure is discarded if there is a more generic one (i.e., a smaller substructure) with the identical summed weight of FP bits. This is done because the more generic substructure in such a case seems sufficient to explain the neuron activation and is more likely to match test compounds.

The FCs are considered for substructure extraction in order of decreasing support (number of compounds represented by the FC) to ensure substructures corresponding to the most generic FCs (highest support) are included. The number of extracted substructures per neuron is limited to a maximum of 200. Moreover, the bits that are already included in the intent of selected FCs are recorded for each neuron. If all bits of a given FC have been included in selected FCs at least once, the FC is directly skipped to accelerate the extraction and to avoid too many very similar substructures. The substructures are then organized into a hierarchical network according to substructure-superstructure relationships to form networks of more generic (smaller) and specific (larger) chemical substructures. This was done to facilitate the matching of test compounds to extracted substructures, see below. This approach of hierarchically organizing chemical substructures is comparable to that used in the self-organizing hypothesis networks (SOHN),^51^ however, here the substructures are organized purely according to substructure-superstructure relationships, whereas, in the SOHN approach, the hierarchical networks are used to analyze structure–activity relationships (e.g., if a certain substructure is mutagenic or not).

### Attribution Methods

The substructures associated with the hidden neurons can be used to assign atom attributions to test compounds in order to provide an explanation for the prediction made by the model. Attribution methods are a common strategy used to highlight atoms in a structure that are important for a QSAR prediction.^19^ As mentioned in the Introduction, these techniques are usually applied to the input features, with IG^20^ an established method that has been applied to a NN trained on Morgan FPs as input.^33^ The approach developed here also uses IG but, in this case, IG is applied to the hidden neurons of the DNN to first indicate the importance of the neuron to the prediction. The importance is then combined with the substructures associated with the neurons that are present in the test compound. A comparable approach to our method (i.e., combining feature visualization with attribution) has been proposed for computer vision models.^52^

In the following, the approach of applying the attribution to the hidden neurons which has been developed here is referred to as IG_hidden, whereas the established approach of applying attribution to the input features is referred to as IG_input. A theoretical background to IG in general is provided below, followed by the details of the attribution methods.

### IG Overview

IG belongs to the gradient-based methods^53^ which assign importance to an input feature by determining its gradient with respect to the model output (i.e., the partial derivative for the feature value of a given instance). Gradient-based methods can only be applied to differentiable models, which include NNs. In the IG method, the gradient of each feature is integrated along a straight line between an input vector *x* and a baseline vector *x*′ (in the case of chemical fingerprints the baseline vector is when all bits are set to zero). The linear path between *x′* and *x* can be described with the term
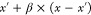
1where β takes values in the range [0,1]. The attribution a for a feature *i* of an instance *x* is computed by
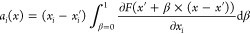
2where *F*() is the NN model. In practice, the integral can be approximated by replacing it with a sum of partial derivatives evaluated at *m* equally spaced steps on the path from *x*′ to *x* as follows:
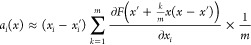
3

A useful property of IG is that the sum of all attributions for a given instance *x* is equal to the difference in the model’s output for *x* and the baseline *x*′.

### IG_Input

**Figure 3:**
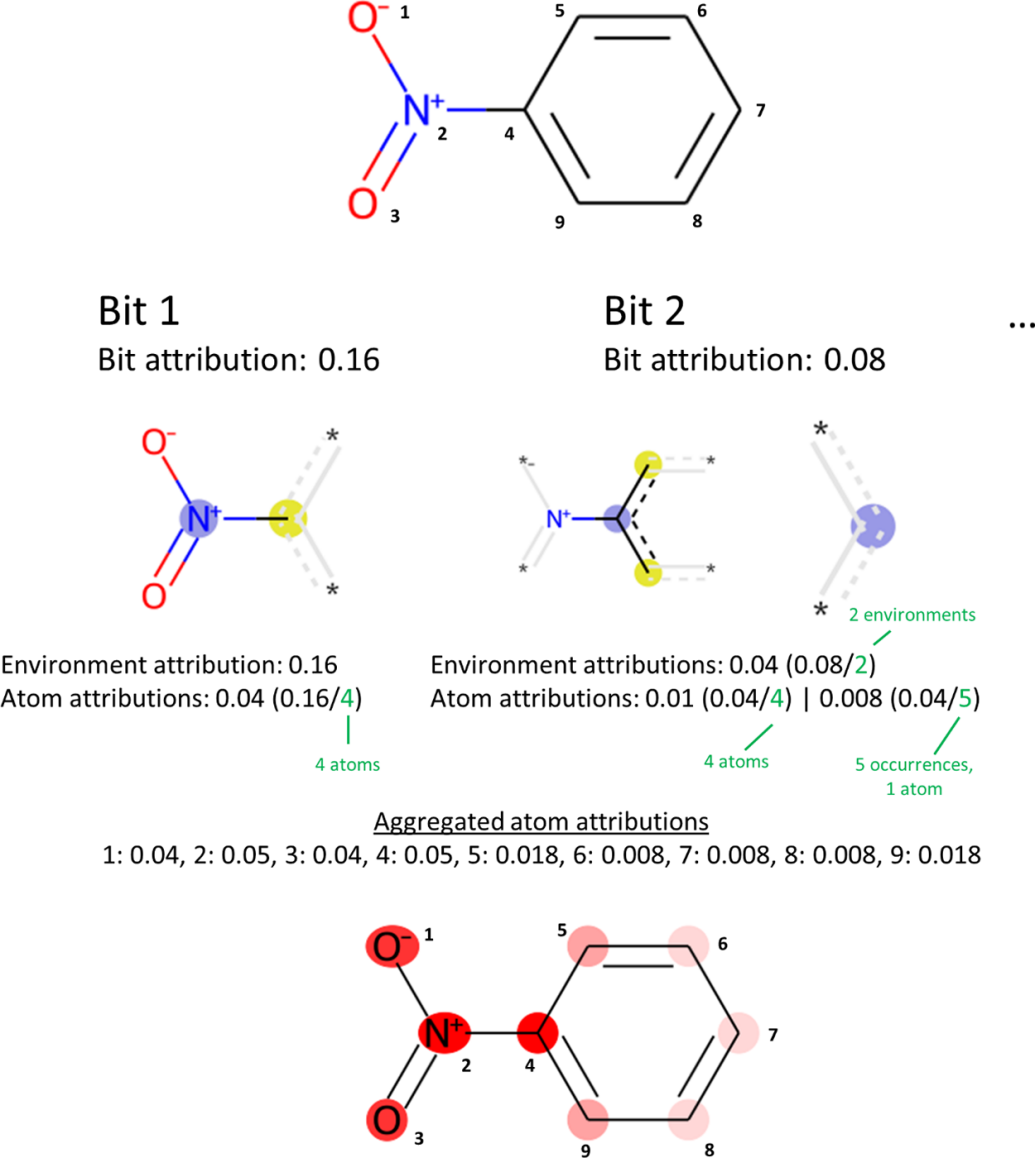
Illustration of IG_input. In this simplified illustration, attributions for two bits (Bit 1 and Bit 2) are depicted. (Note that the mapping between bits and atom environments has been selected for illustration purposes and does not correspond to RDKit’s implementation of Morgan FPs.) Bit 1 belongs to a single atom environment. Therefore, the full attribution for Bit 1 (0.16) is assigned to the environment and is shared equally between all atoms in the environment (1, 2, 3 and 4). A rare case of bit collision is illustrated for Bit 2 where two different atom environments map to the same bit. Therefore, the bit attribution is shared equally between both environments. The first of the two environments contains four atoms and the environment attribution is shared among the respective atoms (2, 4, 5 and 9). The second of the two environments contains just a single atom, but has five occurrences in the compound. The environment attribution is shared between those five atoms (5, 6, 7, 8, 9). To obtain the depiction, the atom attributions obtained from all bits are aggregated. Details of how the highlight colors are obtained are described in the text.

IG determines the importance (positive or negative) of each input feature toward the prediction of a given test compound. In the implementation used here, the attributions obtained for features (i.e., bits of the Morgan FP) are mapped to the atoms in a procedure comparable to the previous study.^33^ First, all-atom environments belonging to a given FP bit are collected. Multiple environments for a given bit may exist due to multiple occurrences of identical environments in a compound or due to bit collisions (i.e., when different environments map to the same position in the bit vector). If necessary, the total attribution for a given bit is shared equally between all environments it is associated with. Then, the attribution assigned to an atom environment is shared equally among the atoms. Atoms may receive multiple attributions due to being in the environments of multiple bits and the attributions may be positive or negative. All attributions for a given atom are summed to obtain the final attribution for the atom. To simplify the calculations, only FP bits with an attribution of at least 1% of the most important feature (positive or negative) are considered. An illustration of the method is provided in Figure 3. The IG_input method was implemented using the *IntegratedGradient* class provided in the Python library Captum (version 0.4.0).^54^

### IG_Hidden

In the IG_hidden method, IG is used to assign importance (i.e., attribution) to each neuron of the hidden layer for the prediction of a test compound and this attribution value may be positive or negative. Then, for each neuron, the substructures associated with it are matched to the test compound, and the neuron’s attribution value is shared between the atoms of the most specific substructures that match the test compound. As described above, each hidden neuron is associated with multiple chemical substructures organized in hierarchical networks. The substructures in the networks are matched to the test compound starting with the most generic substructures. If the test compound does not contain a given substructure, none of its (more specific) child substructures will match. If a test compound does not match any of the substructures extracted for a given neuron, the attribution for that neuron is ignored. This means that the attributions for some of the neurons may not be used to explain the prediction (i.e., will not contribute to the atom coloring which is described below). On the other hand, if multiple substructures are found, the importance of the neuron is shared among them equally. Two different schemes were investigated to map the attributions of a substructure to individual atoms. In the first, the attribution for a given substructure is shared equally by all atoms of the substructure (as is done for environments in the IG_input method). In the second case, in addition to sharing the attribution among the atoms, different weights are considered for the atoms forming a substructure based on the weights the individual FP bits have for the neuron. The weights are intended to indicate the relative importance of each atom of the substructure with respect to the neuron activation in order to make the eventual model explanations more accurate. For a given substructure, there is a set of associated FP bits each with a corresponding weight (network weight from input neuron to the hidden neuron). The weights assigned to the atoms of the substructure are proportional to the summed weight of FP bits that the atom is associated with. The values obtained for each atom are scaled so that the weights of all atoms of a substructure sum to 1. Overall, model explanations were slightly better when the weights were used and therefore the results shown here are based on the weighted contributions only.

**Figure 4:**
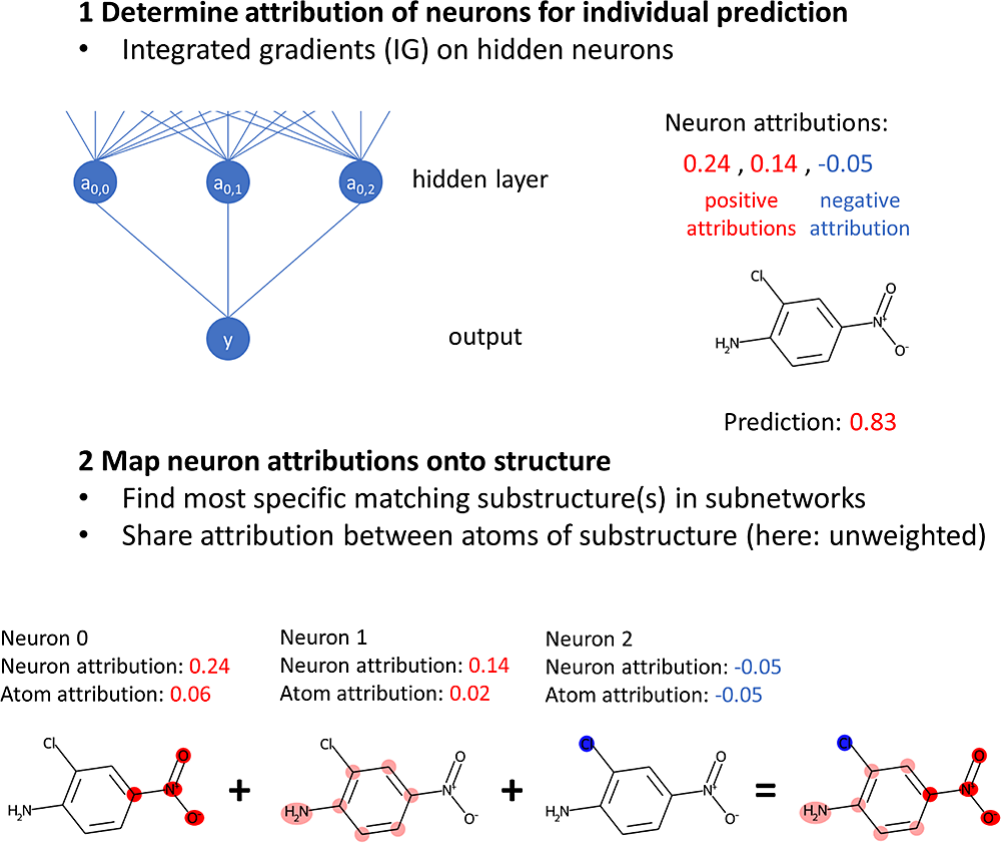
Illustration of IG_hidden. In the first step, an attribution (positive or negative) is determined for each hidden neuron for the test compound. Then, the neuron attributions are converted to atom attributions using matching substructures. In this case, one matching substructure is found for each neuron. The substructure for the first neuron is a nitro group with an aromatic carbon (four heavy atoms). Therefore, the neuron attribution is divided by 4 and an atom attribution of 0.06 is obtained. The same procedure is applied to the other neurons’ attributions. Notably, the attribution for neuron 2 is negative, hence the blue coloring. Details for the atom coloring are provided in the text. In this case, no weighting has been applied to the atoms of a substructure to simplify the illustration. The rightmost structure contains atom colorings aggregated from the individual neurons’ atom attributions.

To obtain a model explanation, the steps described above are repeated for all hidden neurons. The general principle of the IG_hidden method is illustrated in Figure 4 for a simplified case of three hidden neurons. An attribution (positive or negative) is assigned to each neuron and for each neuron the matching substructures for the test compound are found and the neuron attribution value is shared across all atoms of the substructure. This is repeated for each neuron and the atom attributions are summed to give a final model explanation. In the case shown, no weights were used for the atoms of extracted substructures, for simplicity. As for IG_input, the Python library Captum was used (here: *LayerIntegratedGradient* class).

### Depiction of Atom Attributions

Atom attributions for individual compounds are depicted using a color map. Positive attributions (contributing to a toxic prediction) are highlighted in red, while negative attributions (contributing to a nontoxic prediction) are highlighted in blue. Neutral atoms (attribution = 0) are not highlighted (white “highlight”). To make the coloring between different compounds comparable, colors are scaled according to the maximum atom attribution observed in a data set, which may be positive or negative. The maximum atom attribution receives full color intensity and all atoms of the compounds in the data set are assigned colors relative to this maximum. The color intensity for individual atoms is assigned by interpolating in RGB color space. To obtain better discrimination of atoms in the lower range of attributions, the maximum color intensity is assigned to all atoms with attributions at least 70% of the maximum, which is consistent with a previous study.^55^ Separate scales are used for IG_input and IG_hidden due to the observation that larger atom attributions are generally obtained for IG_input. The reason for this mostly seems to be that attributions for IG_hidden are ignored when no matches are found for a given neuron. As a result, color intensities between IG_input and IG_hidden are not directly comparable.

### Evaluation of Atom Attributions

The quality of atom attributions for individual predictions was evaluated using the data set based on Derek Nexus labels (i.e., structural alerts) as ground truth. The output from Derek Nexus is a report of which atoms are responsible for an alert being fired. For a given compound, the ground truth is defined as the union of all atoms responsible for all alerts that are fired. The concordance of atom attributions output by IG_hidden with the ground truth for a compound with a positive prediction of toxicity was measured using ROC-AUC.^56^ The atoms are ranked on their attribution and at each threshold the TPR (true positive rate, i.e., recall) and FPR (false positive rate, i.e., 1-specificity) with respect to the ground truth are recorded and the area under the obtained ROC curve is determined. Note that the attribution ROC-AUC cannot be computed for compounds where all atoms form the ground truth for toxicity because no FPR can be computed.

Naturally, attribution AUC values can only be determined for compounds matching an alert (actual positives). These cases can be further discriminated into TPs (true positives: correctly predicted as positive) and FNs (false negatives: incorrectly predicted as negative). For an FN compound, the explanation cannot be expected to match the true cause of toxicity, since the model did not predict the compound as toxic. This is a mistake made by the NN. However, the objective of this analysis is to evaluate the performance of the attribution method. Therefore, attribution AUC scores were only computed for TP compounds and the distribution of attribution AUC scores was calculated. This process was repeated for the TP compounds using the IG_input method in order to compare the performance of IG_hidden with the more established attribution method.

A deeper understanding of the performance of attribution methods can be gained by analyzing attribution AUC scores obtained for specific alerts. It may be that an attribution method performs very well for some alerts, but poorly for others. For this analysis, only compounds matching a single alert were considered. Two alerts (Alert39 and Alert87) were almost always found co-occurring with the alert for alkylating agents (Alert53) and in this case, they were added to the support set for Alert53 to be included in the analysis. Then, for each alert, the mean attribution AUC across compounds matching this alert was computed.

## Results

### Model Performance

Model classification performance on both validation and test sets is reported in Table 3 using several metrics for the three different models: the single-layer model trained on experimental Ames labels; the single-layer model trained on Derek labels; and the two-layer model trained on Derek labels. Good performance is achieved for the model trained on experimental Ames labels with an accuracy of above 0.8, an ROC-AUC score of around 0.9, and an MCC score above 0.6 on both validation and test sets. While optimizing model performance was not the focus of this work, we note that our reported scores are in a comparable range to other recent models reported in the literature that predict Ames mutagenicity.^57,58^ Even better performances were observed for models trained on Derek Nexus labels. This may be because it is less challenging to predict well-defined chemical rules that are not prone to experimental uncertainty. For the Derek Nexus labels, the two-layer model slightly outperformed the single-layer model.

**Table 3:** Model Performances (Validation/Test)

	accuracy	ROC-AUC	precision	recall	MCC
Ames model (one layer)	0.825/0.806	0.905/0.890	0.817/0.816	0.837/0.813	0.650/0.611
Derek Nexus label model (one layer)	0.903/0.900	0.970/0.965	0.889/0.897	0.929/0.922	0.807/0.799
Derek Nexus label model (two layers)	0.918/0.906	0.977/0.964	0.918/0.932	0.923/0.892	0.836/0.812

### Exploration of Chemical Features Learned in Hidden Neurons

**Figure 5:**
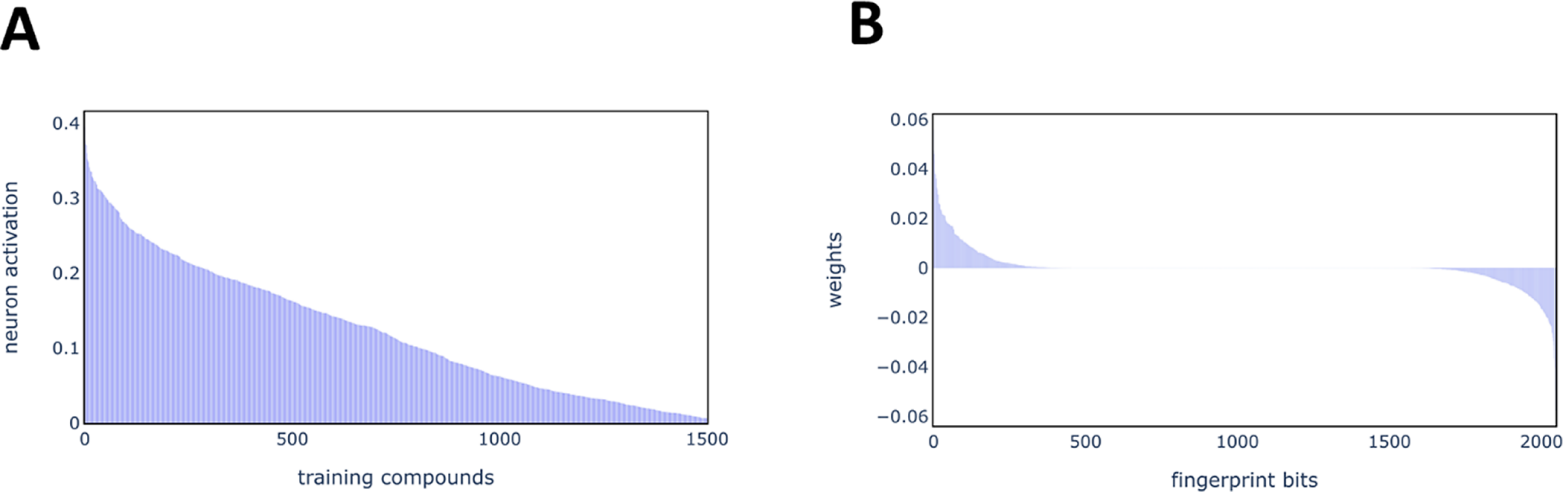
Training compound activations and learned weights for neuron 1–153. (A) Distribution of activations for training compounds sorted in descending order (Top-1500 compounds shown). (B) Distribution of learned weights connecting input neurons to neuron 1–153.

**Figure 6:**
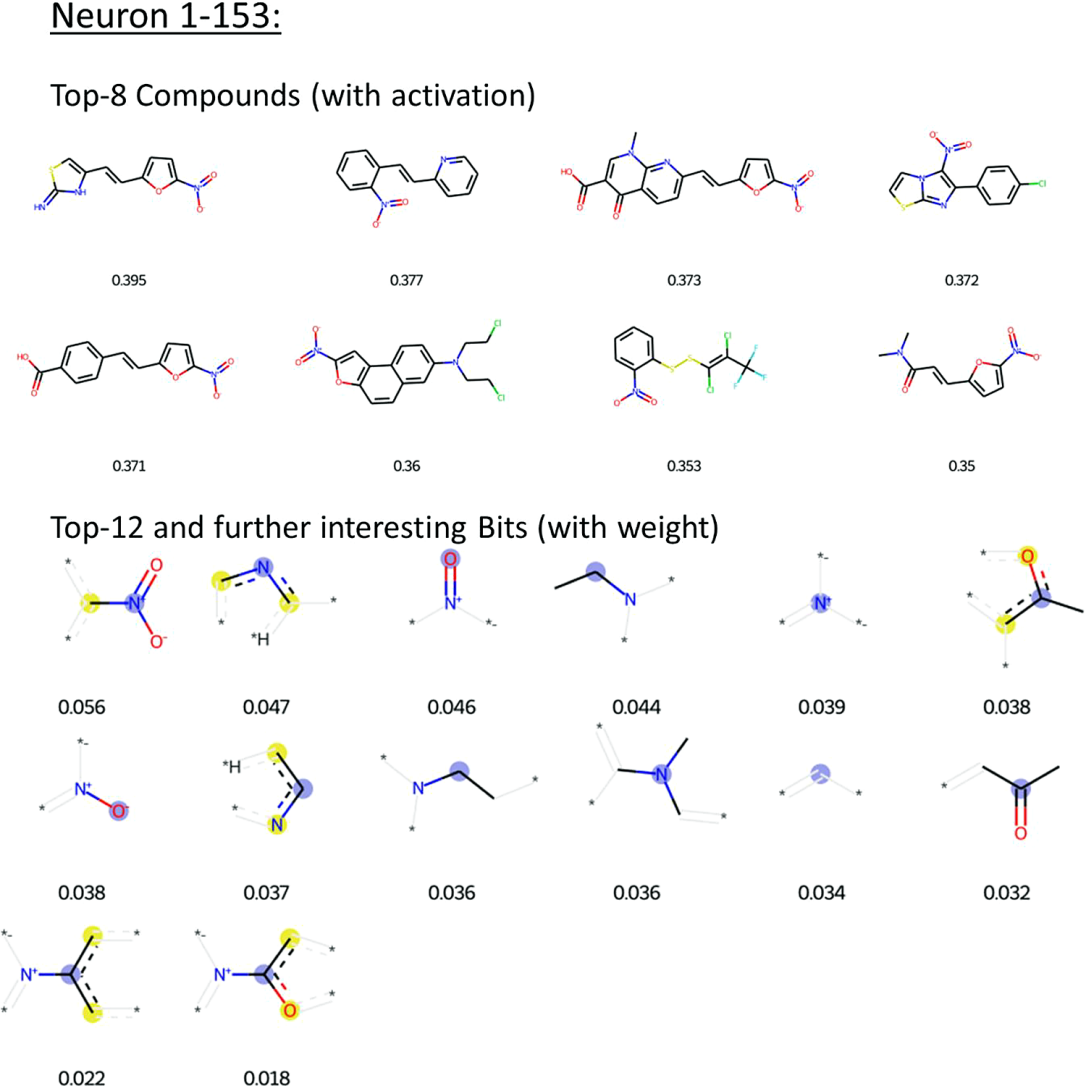
Most relevant training compounds and FP bits for neuron 1–153. Shown are the Top-8 training compounds (strongest activation) and the Top-12 FP bits (highest weights), as well as two further bits linked to the aromatic nitro group (bottom row of lower panel).

A preliminary investigation was conducted as a proof of concept to explore whether the activation of hidden neurons can be linked to the presence of certain chemical features. This used the single hidden layer NN trained on the Ames data set. Two sources of information were considered: the training compounds most strongly activating a given neuron; and the Morgan FP bits associated with the neuron that have high learned weights. The distribution of training compound activation values for neuron 1–153 can be seen in Figure 5 alongside the distribution of weights for all input fingerprint bits. The strongest activation is at 0.395 and many training compounds have activations of comparable magnitude (e.g., 312 training compounds have activation values >0.2). The weights for the FP bits range between 0.056 and −0.057 with many bits having a weight close to zero. The eight training compounds that most strongly activate the neuron and the FP bits with the highest learned weights are shown in Figure 6.

All of the Top-8 compounds for neuron 1–153 contain an aromatic nitro group, which is a known toxicophore for mutagenicity. The nitro group is attached to different aromatic rings, namely phenyl, furane, and bi- or tricyclic systems. When inspecting the Top-12 bits (top two rows of the lower panel in Figure 6), several can be identified that are bits linked to the aromatic nitro group, including those with the highest and third highest weights. Moreover, two further bits indicative of an aromatic nitro group but which are outside the Top-12 bits are shown in the bottom row. The weights for these bits, while lower than those in the Top-12, still make an appreciable contribution to the neuron activation observed for aromatic nitro compounds. From these observations, it can be concluded that neuron 1–153 detects aromatic nitro compounds. Notably, it is connected to the output neuron with a positive weight (0.05), indicating that its activation increases the probability of a mutagenic prediction being made by the NN.

The Top-8 compounds and Top-12 bits for further example neurons are shown in the Supporting Information. These include examples of neurons that detect epoxides (Figure S1, neuron 1–43), polycyclic aromatic hydrocarbons (Figure S2, neuron 1–180), both azides and acridine (Figure S3, neuron 1–69) as well as a further neuron that detects aromatic nitro compounds (Figure S4, neuron 1–71). These examples show that a single neuron may detect multiple relevant chemical features and conversely, different neurons may detect the same or similar chemical features.

It can be concluded that inspecting training compounds with high activation and bits with high weights may provide some insights into the chemical features a hidden neuron responds to. However, a manual analysis for all hidden neurons would be cumbersome and subject to human bias. Moreover, focusing on a small subset of compounds and FP bits might lead to relevant chemical features that cause neuron activations of medium strength to be missed. Therefore, the automated method for identifying substructures linked to activation of hidden neurons was developed as described in the Methodology. The results of applying this approach are described below.

### Atom Attributions for Models Trained on Derek Nexus Alerts

The automated method for identifying substructures that cause activation of hidden neurons was evaluated both globally (considering the entirety of extracted substructures) and locally (considering individual predictions).

#### Global Analysis

The NN trained on Derek Nexus alert labels was used for this evaluation, where the cause of labels is known. In total, 39,164 substructures were extracted across all hidden neurons of the network (identical substructures may be extracted for different neurons). The median number of extracted substructures per hidden neuron was 138. The extracted substructures cover all but one of the Derek Nexus alert structures: of the 102 Derek Nexus alerts contained in the training data set, superstructures for 101 of them are among the extracted substructures across all hidden neurons.

**Figure 7:**
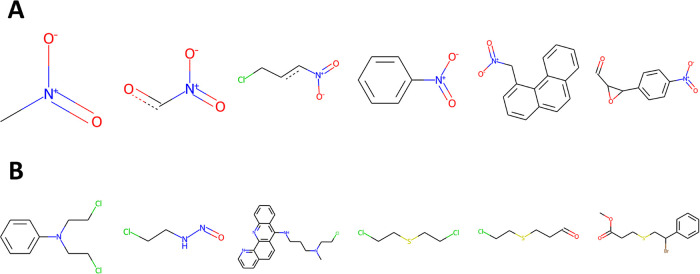
Example extracted substructures. (A) Aromatic nitro alert. (B) Nitrogen or sulfur mustard alert.

Some extracted substructures are shown for two selected Derek Nexus alerts, in Figure 7 (panel A: aromatic nitro alert; panel B: nitrogen or sulfur mustard alert). In total, 4999 substructures containing aromatic nitro were extracted from 213 different neurons, and 327 substructures containing a nitrogen/sulfur mustard group were extracted from 166 different neurons. This suggests that chemical features related to a specific alert are detected by many neurons across the network. However, a substructure may contain more than one relevant chemical feature (see below).

Extracted substructures may be small (e.g., a nitro group attached to a single aromatic carbon atom) or quite large (e.g., a nitro group attached to a larger ring system). Notably, a substructure may contain more than one chemical group associated with mutagenicity. For instance, the rightmost structure in the top row of Figure 7 contains an aldehyde and an epoxide group in addition to the aromatic nitro. The bottom row shows that both nitrogen and sulfur mustard groups were among the extracted substructures. In the majority of cases, the mustard group is formed with one or two chlorine atoms, yet the rightmost substructure contains a bromine atom. Overall, this shows that the extracted substructures may cover different variants of a particular alert.

#### Local Analysis

**Figure 8:**
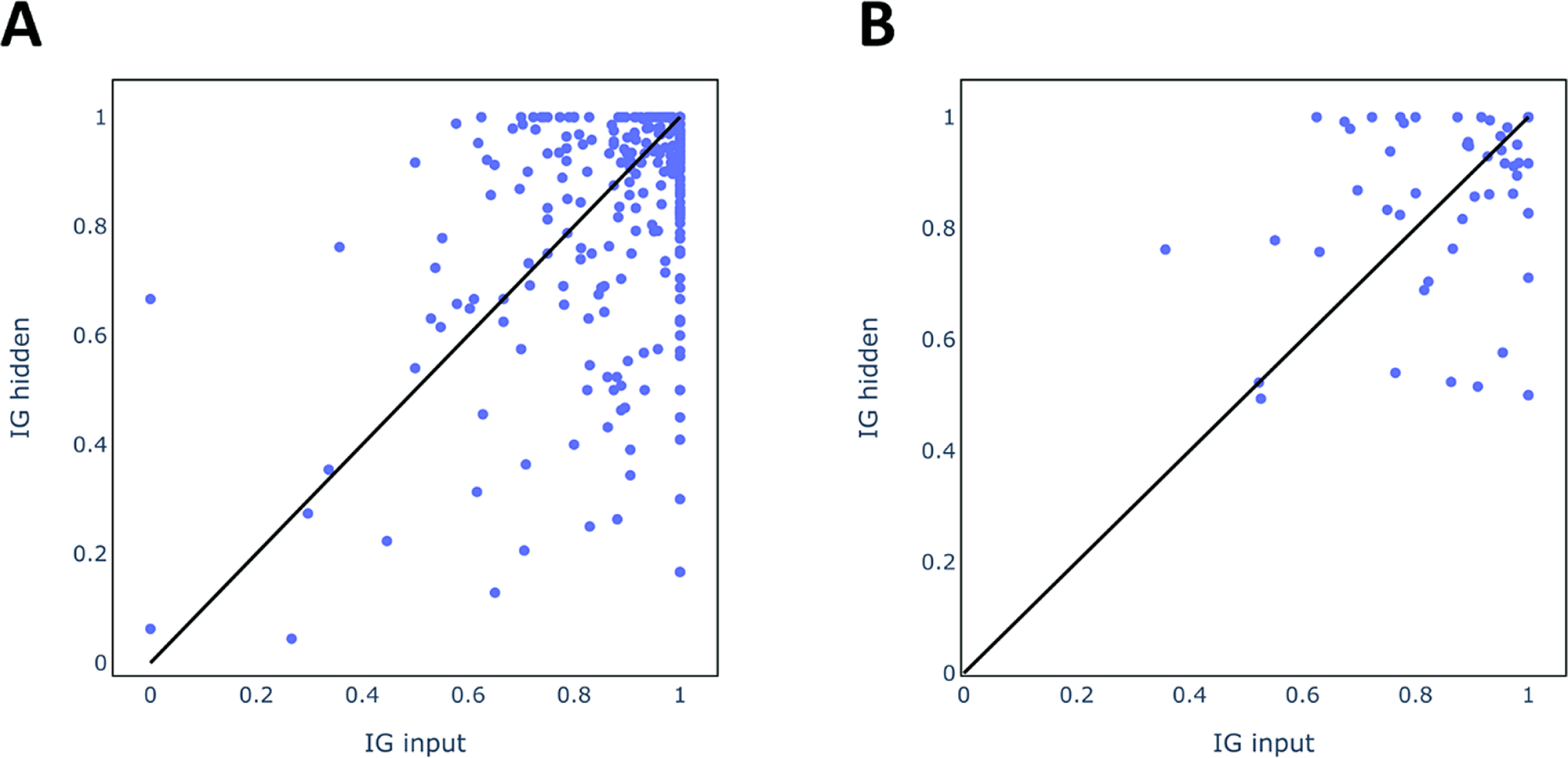
Attribution AUC scores of individual compounds (A) and alerts (B).

The IG_hidden method was applied to compounds in the validation set (and later the test set) to evaluate if the extracted substructures may be used to explain individual predictions made by the NN. The results were compared with the established IG_input approach where IG is used to determine the importance of input features which are subsequently mapped to test compounds. The results in Table 4 show: the quality of model explanations as median attribution AUCs; the proportions of compounds with attribution AUC of ≥0.8; the median alert attribution AUCs; and the proportion of alerts with attribution AUCs of ≥0.8 for the validation set. Moreover, in Figure 8 attribution AUCs of individual compounds (A) and alerts (B) are compared. IG_hidden was also used to color the atoms of a validation compound according to the substructures extracted from the highly activating neurons as described above.

**Table 4:** Evaluation of Model Explanations on the Validation Set^a^

	median AUC	AUC ≥ 0.8	median alert AUC	alert AUC ≥ 0.8
IG_input	0.964	0.817	0.894	0.692
IG_hidden	0.935	0.727	0.903	0.712

a Shown are the median attribution AUC across TP compounds; the proportion of compounds with an attribution AUC of ≥0.8; the median alert attribution AUC; and the proportion of alerts with an attribution AUC ≥ 0.8.

While both approaches achieve good explanations for the majority of compounds (AUC of at least 0.8 for 82 and 73% for IG_input and IG_hidden, respectively), IG_input outperforms IG_hidden overall when evaluating individual compounds. Figure 8A shows the AUC scores for individual compounds where for some compounds IG_input clearly outperforms IG_hidden, whereas the opposite is the case for other compounds. For a few compounds, IG_hidden provides very poor scores (AUC ≤ 0.5). However, this is a biased evaluation as some alerts are much more frequent in the data set and hence the distribution of attribution AUC scores is dominated by a few alerts. Figure 8B shows the average explanation scores for individual alerts and can be considered a more robust comparison, as all alerts are considered equally important. In this evaluation scheme, neither of the approaches is clearly superior and, as for individual compounds, each method outperforms the other for a subset of alerts. Similar conclusions can be drawn when evaluating the test set (see Figure S5).

**Figure 9:**
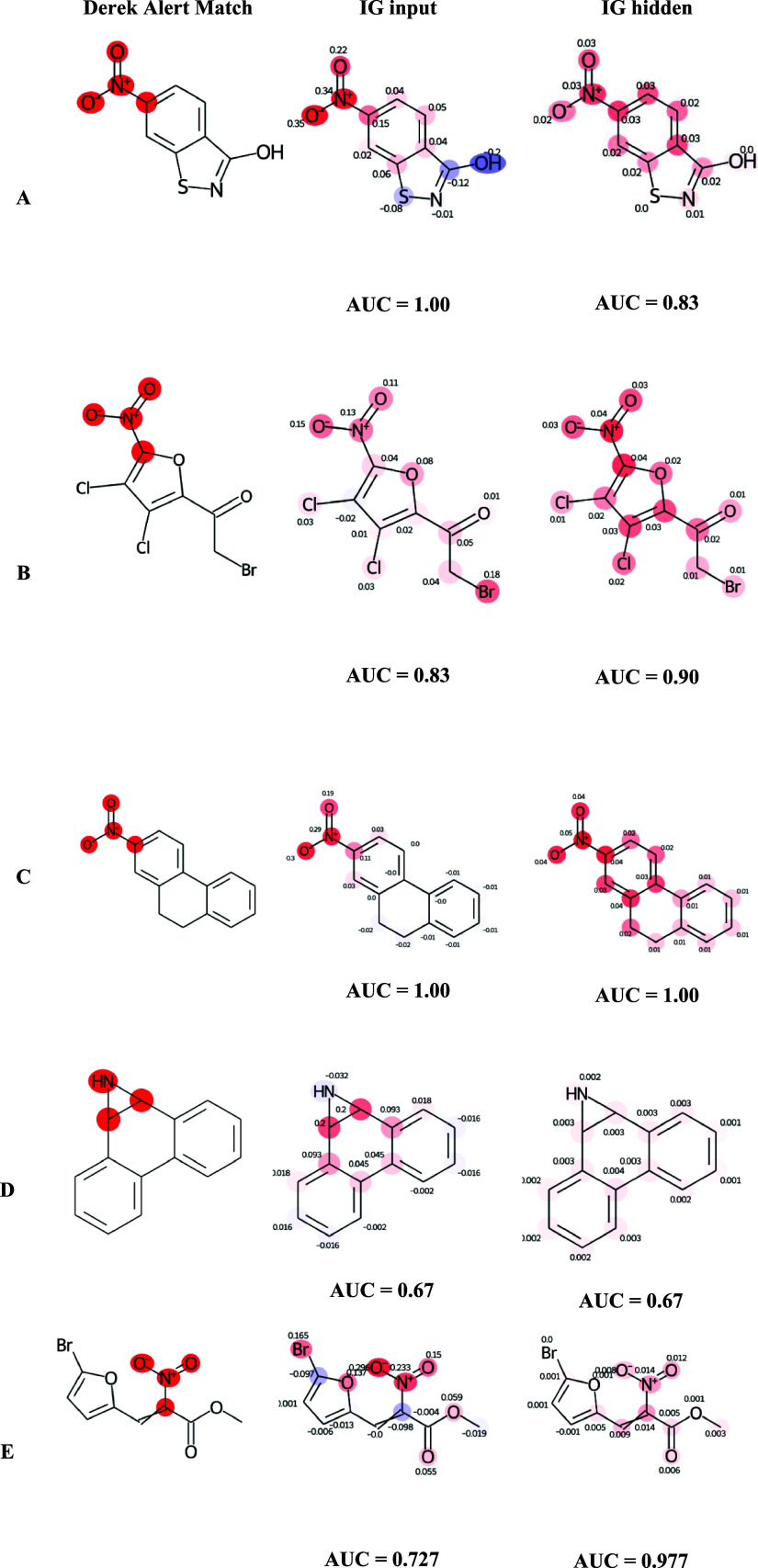
Comparison of atom attributions for individual compounds (A to E, respectively). Shown are compounds with Derek Nexus alerts highlighted (first column); atom attributions along with the corresponding attribution AUC for IG_input (second column) and IG_hidden (third column).

Model explanations obtained by IG_input and IG_hidden for some example compounds are provided in Figure 9 and are compared with the “ground truth” as presented by Derek Nexus alerts. An AUC of 1 will be achieved if all the atoms reported in the Derek Nexus match are given the highest contribution. An AUC of 1 can also be obtained where additional atoms show positive attributions provided that their values are lower than those of the atoms in the Derek Nexus alert.

For the first compound (Figure 9A), a perfect attribution AUC score of 1 was achieved by IG_input which means that all atoms belonging to the Derek Nexus alert (which forms the ground truth) were assigned higher attribution values than all remaining atoms. On the other hand, IG_hidden received a lower AUC of 0.83 due to equal contribution from atoms in the phenyl ring not covered by the Derek Nexus alert. The AUC score depends on the definition of the alert and in this case, highlighting the complete phenyl ring may still be considered a correct explanation even though it is not included in the atom match list for the Derek Nexus alert which conveys mutagenicity of `Aromatic Nitrò compounds, more generally.

In the example in Figure 9B, the AUC for the IG_input approach is lower than the AUC of the IG_hidden approach. The IG_input method receives a lower AUC due to the relatively high contribution of the bromine atom, whereas, although the bromine atom is also highlighted in the IG_hidden method, it has a lower ranking than the other highlighted atoms. In this example, even though the IG_hidden approach has a higher AUC, subjectively the IG input method gives a clearer picture with minimal contribution from the non nitro atoms.

In the example in Figure 9C, the AUC is 1 in both cases with the Aromatic nitro atoms identified by the Derek Nexus alert having the highest ranks, however, the IG_hidden method attributes greater contribution to atoms in the aromatic ring that are not explicitly covered by the Derek Nexus aromatic nitro alert, compared to IG_input.

In the example in Figure 9D, for the IG_hidden method, the contribution is shared across all atoms in the fused ring system resulting in low intensity for all atoms. The IG_input method yields a high intensity around the aziridine group, however, it has attributed a negative score to the nitrogen. This example demonstrates a limitation of IG_hidden where low intensity is seen for a large substructure (the total color intensity is divided among atoms).

In the example in Figure 9E, the IG_hidden method yields a higher AUC than the IG_input and is a closer match to the ground truth. The IG_input method has identified unrelated atoms in the neighboring ring which are ranked more highly than the attachment point of the nitro group.

**Figure 10:**
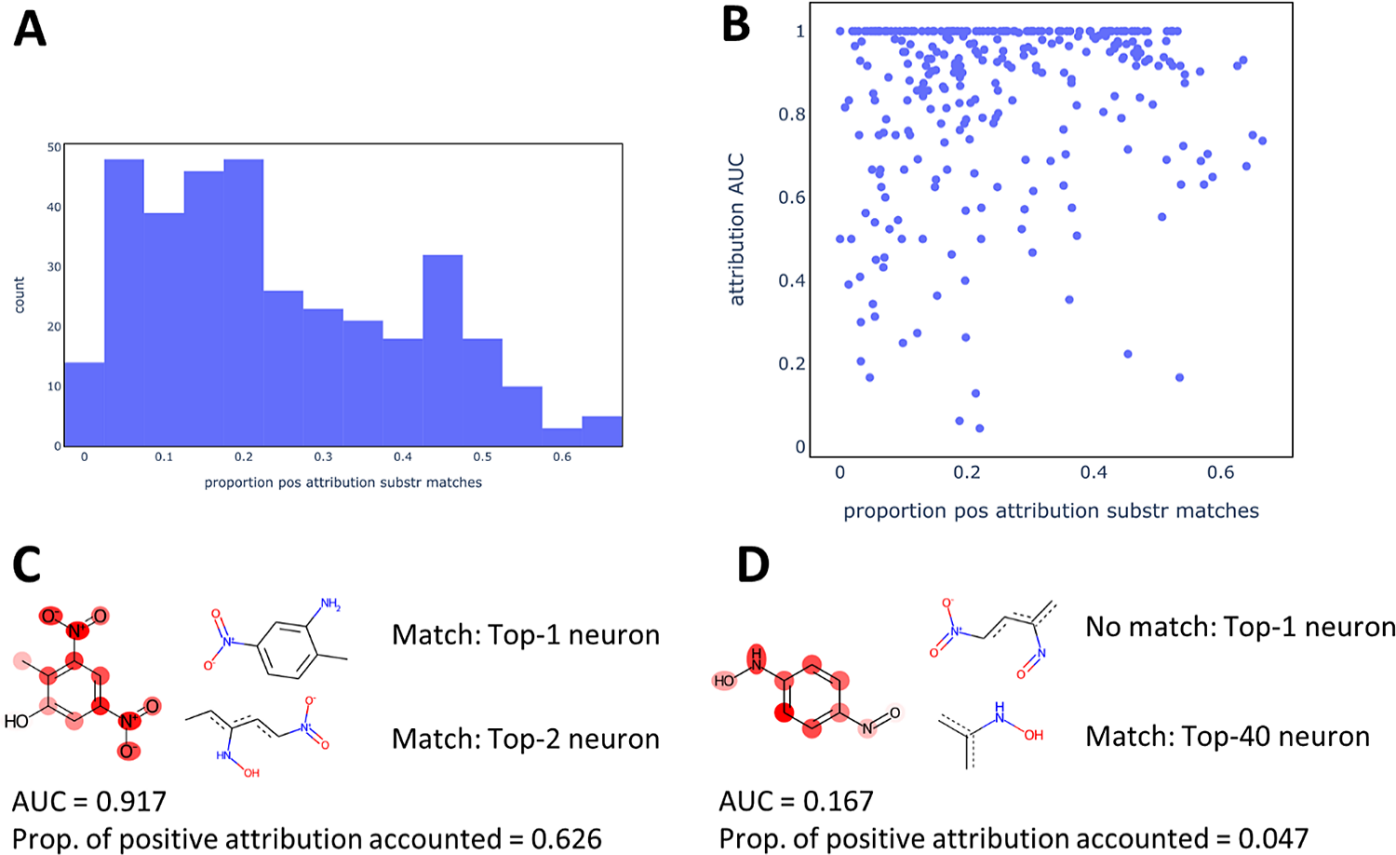
Analysis of positive attributions accounted for in model explanations by IG_hidden. (A) Histogram showing proportions of positive attributions accounted for in TP compounds of the validation set. (B) Scatter plot showing proportions of positive attributions and attribution AUC values for individual TP compounds in the validation set. (C) Example with high proportion of positive attribution accounted for. Ground truth: nitro groups and the attached aromatic carbon atoms. (D) Example with low proportion of positive attribution accounted for. Ground truth: nitroso group and hydroxylamine as well as the attached aromatic carbon atoms.

When explaining the prediction of a compound using IG_hidden, it may be that the compound does not match any of the substructures extracted for a given neuron. In that case, the attribution for this neuron does not contribute to atom coloring (see Methodology). Figure 10A shows the proportions of positive attributions that are accounted for by the IG_hidden explanation model for the validation compounds.

For many TP compounds, only a small proportion of positive attribution was accounted for in the obtained model explanations. For many compounds, this value was below 0.2, while the highest observed proportion across all TP compounds was 0.666. However, the magnitude of the proportions is not correlated with the quality of model explanations (Figure 10B). High AUC scores were obtained for low, medium, and high proportions of accounted-for attributions. Two example compounds are shown in Figure 10C and D, respectively. The compound in Figure 10C is an example where a high proportion of positive attributions is accounted for. As is shown, the two neurons with the highest positive attributions are both associated with substructures matching the test compound. On the other hand, for the compound in Figure 10D, a very low proportion of positive attribution is accounted for (0.047). In particular, the neuron with the 40th largest proportion is the first neuron for which a matching substructure (aromatic hydroxylamine) has been extracted. This is associated with a poor model explanation for this compound (AUC = 0.167). Also shown is an extracted substructure for the neuron with the highest attribution for this compound. This substructure contains an aromatic nitroso group (like the test compound), but also a nitro group and hence does not match. This suggests that the neuron is activated by the presence of the nitroso group, but the absence of a matching substructure associated with the neuron (e.g., a generic aromatic nitroso), means that this information is not used in the model explanation by IG_hidden. This is an example where the extraction of more generic substructures could have improved the quality of model explanations.

### Atom Attributions for Models Trained on the Experimental Ames Data

Next, the IG_hidden method was evaluated using the model trained on experimental Ames labels, which is how QSAR models are used in practice. As in the previous experiment, the Derek Nexus alert atoms are used as ground truth, and the quality of the model explanations was evaluated on individual compounds and averaged for alerts. For this evaluation, the validation and test set were pooled. Median scores are reported in Table 5 and scatter plots contrasting individual scores for IG_input and IG_hidden are shown in Figure S6. All the median scores for IG_input are slightly higher than for IG_hidden, yet, as before, there are subsets of compounds where IG_hidden provides more accurate model explanations. Overall, the scores are somewhat lower compared to the model trained on Derek Nexus labels. This was expected, as the model in this case was not trained on the ground truth used to evaluate model explanation. Nonetheless, it can be concluded that both IG_input and IG_hidden provide good model explanations for a majority of compounds.

**Table 5:** Evaluation of Model Explanations for the Model Trained on Experimental Ames Labels^a^

	median AUC	AUC ≥ 0.8	median alert AUC	alert AUC ≥ 0.8
IG_input	0.905	0.726	0.848	0.574
IG_hidden	0.883	0.640	0.814	0.537

a Shown are the median attribution AUC across TP compounds, the proportion of compounds with an attribution AUC of ≥0.8, the median alert attribution AUC, and the proportion of alerts with an attribution AUC ≥ 0.8.

**Figure 11:**
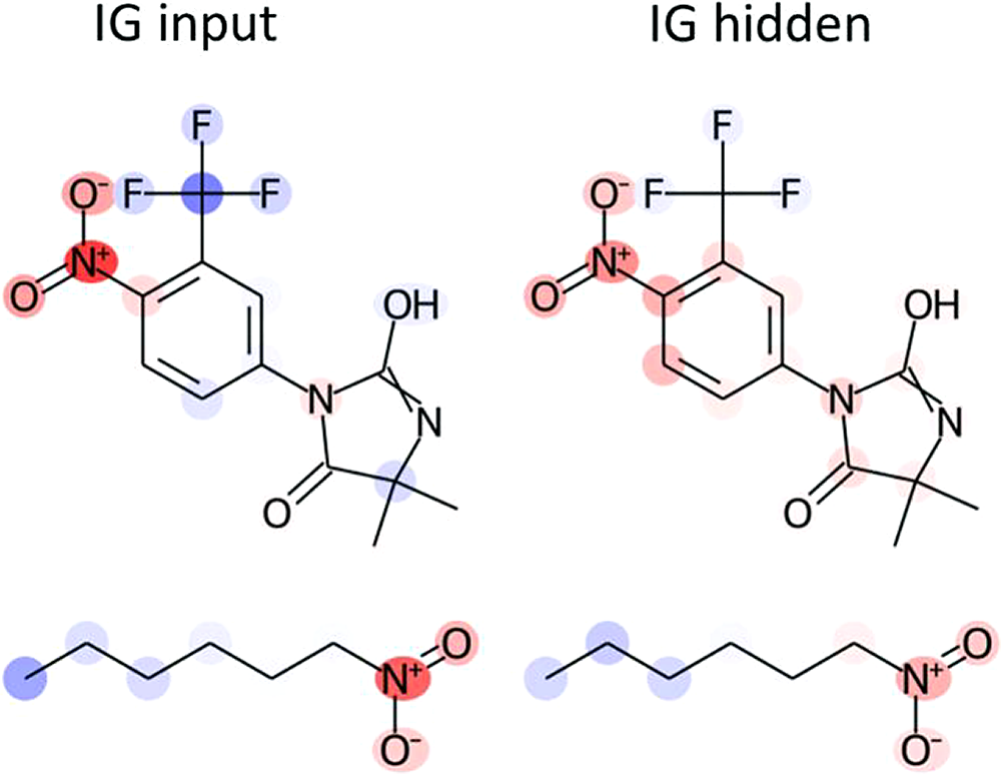
Example explanations for Ames negative predictions.

Having established that the methods are successful in identifying substructures associated with positive predictions, they were then applied to attempt to explain negative predictions made by the NN. Model explanations for two example compounds that were correctly predicted as Ames negative are presented in Figure 11.

The first example shows an aromatic nitro compound with a deactivating trifluoromethyl group in the ortho position. This compound is negative in the Ames Test, as the trifluoromethyl group withdraws electron density from the aromatic system thereby reducing the stability of the nitrenium ion responsible for its mutagenic potential.^59^ This compound was correctly predicted as negative by the model and both IG_input and IG_hidden assign negative attributions to the trifluoromethyl group, although this is more pronounced in IG_input. In the second example, negative attribution is assigned to the alkyl chain by both approaches, while the nitro group is assigned positive attribution. Aliphatic nitro compounds are not listed as toxicophores for mutagenicity in the public ToxAlert database.^35^ It can be concluded that explanation methods may correctly assign negative attribution to chemical features that reduce or eliminate the mutagenic potential of compounds. However, and as was the case for infrequent toxicophores in a data set, the IG_hidden method may fail to extract relevant substructures for some of the relevant neurons resulting in pale coloring or a total miss of deactivating features. It is to be expected that evaluating negative (nonmutagenic) model explanations is more difficult, as they may result either from the absence of mutagenic features or the presence of deactivating features, which are generally less well understood than toxicophores for mutagenicity.

### Exploration of Deep Neural Networks

All experiments so far were applied to NNs with a single hidden layer to demonstrate the validity of the approach for a relatively simple model. In practice, DNNs (i.e., more than one hidden layer) may achieve higher prediction scores than those consisting of a single layer. Therefore, the IG_hidden approach was applied to the two-layer neural network trained on the Derek Nexus alert labels. First, the substructure extraction method was applied to the first layer of the network and the extracted substructures were used to explain predictions made by the model. The quality of the explanations obtained by IG_hidden was evaluated in the same manner as for the simpler models and compared to the IG_input method. The AUC scores on the validation set are reported in Table 6.

**Table 6:** Evaluation of Model Explanations for the Two-Layer Model^a^

	median AUC	AUC ≥ 0.8	median alert AUC	alert AUC ≥ 0.8
IG_input	0.984	0.841	0.907	0.714
IG_hidden	0.938	0.725	0.906	0.735

a Shown are the median attribution AUC across TP compounds, the proportion of compounds with an attribution AUC of ≥0.8, the median alert attribution AUC, and the proportion of alerts with an attribution AUC ≥ 0.8.

Overall, the scores are similar to those for the single hidden layer NN. IG_input achieved higher scores when considering the attribution AUC on individual compounds (median 0.984 vs 0.938), while the performance is nearly identical when considering the average scores for alerts (median 0.907 vs 0.906). As for the one-layer NN, IG_input and IG_hidden each perform better on different subsets of compounds and alerts (see Figure S7). It was hence shown that IG_hidden can be applied to the first hidden layer of a two-layer NN to achieve good explanations for a majority of compounds.

**Figure 12:**
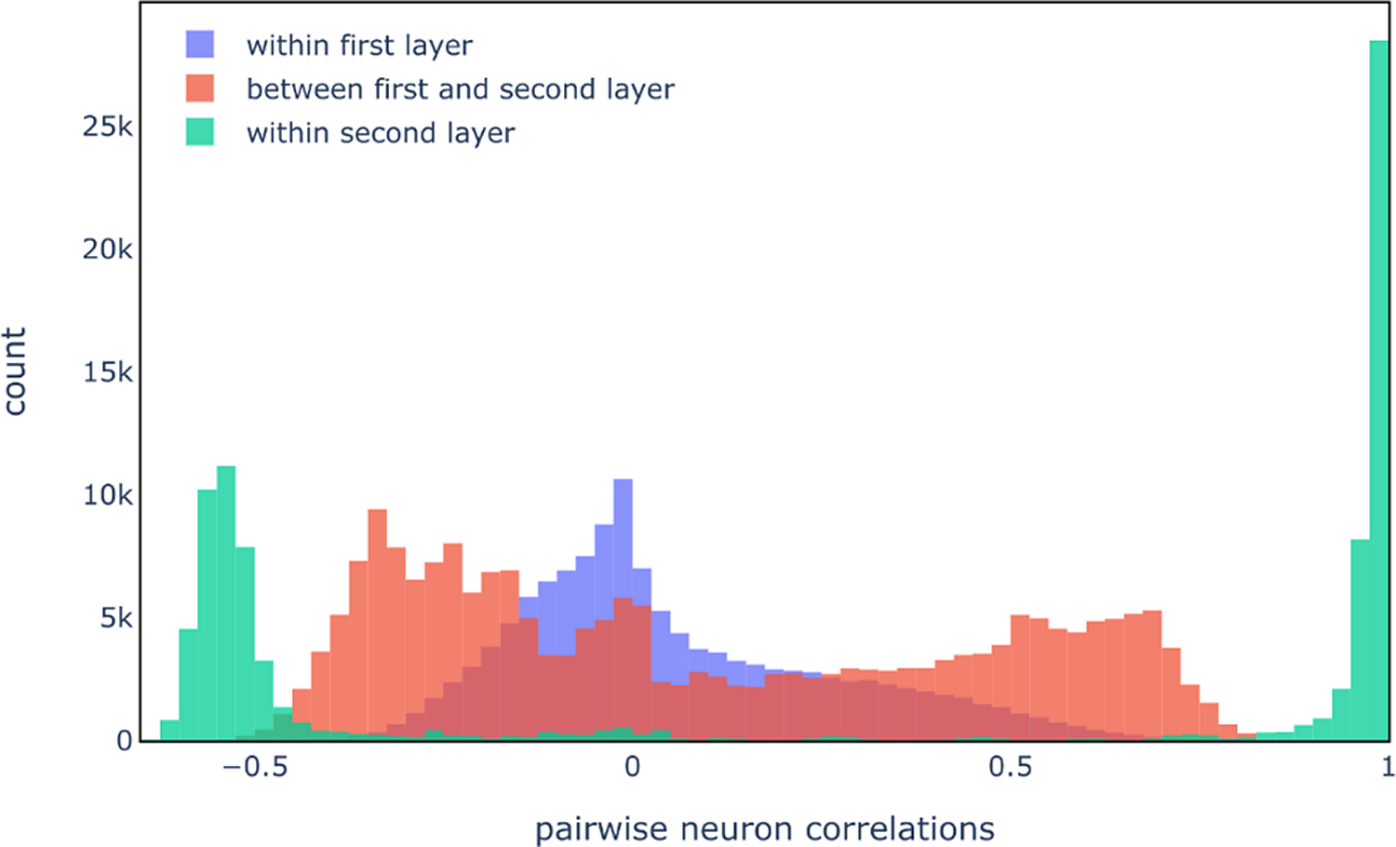
Correlation analysis of hidden neurons. Pairwise correlations are grouped into: pairs of neurons in the first layer (“within first layer”—blue; pairs of neurons in the first and second layer ('between first and second layer'—red); and pairs of neurons in the second layer (“within second layer”—green). The histogram of pairwise correlations for each group is shown. Correlations of neurons with themselves were ignored.

Next, neurons of the second hidden layer were investigated. Before attempting to extract substructures from these neurons, their role in the NN was investigated. First, the pairwise correlation of compound activation between hidden neurons was investigated. A positive correlation indicates that the pairs of neurons are activated by the same compounds. The distribution of pairwise correlations (i) within the first hidden layer, (ii) between the first and second hidden layer, and (iii) within the second hidden layer are shown in Figure 12.

Neuron pairs within the first hidden layer have mostly no or very little correlation (blue histogram). This suggests that there is a diversity in the chemical features that are detected by different neurons in the first hidden layer. The existence of moderately strong correlations between neurons in the second and first hidden layer (red histogram) suggests that neurons in the second hidden layer, to some extent, detect similar chemical features to those detected in some of the first hidden layer. However, the vast majority of neuron pairs within the second hidden layer (green histogram) either have a strong positive correlation (>+0.9) or a moderately strong negative correlation (<−0.5). This means that many of the neurons in the second layer detect the same chemical features and that there is little diversity between the neurons.

**Figure 13:**
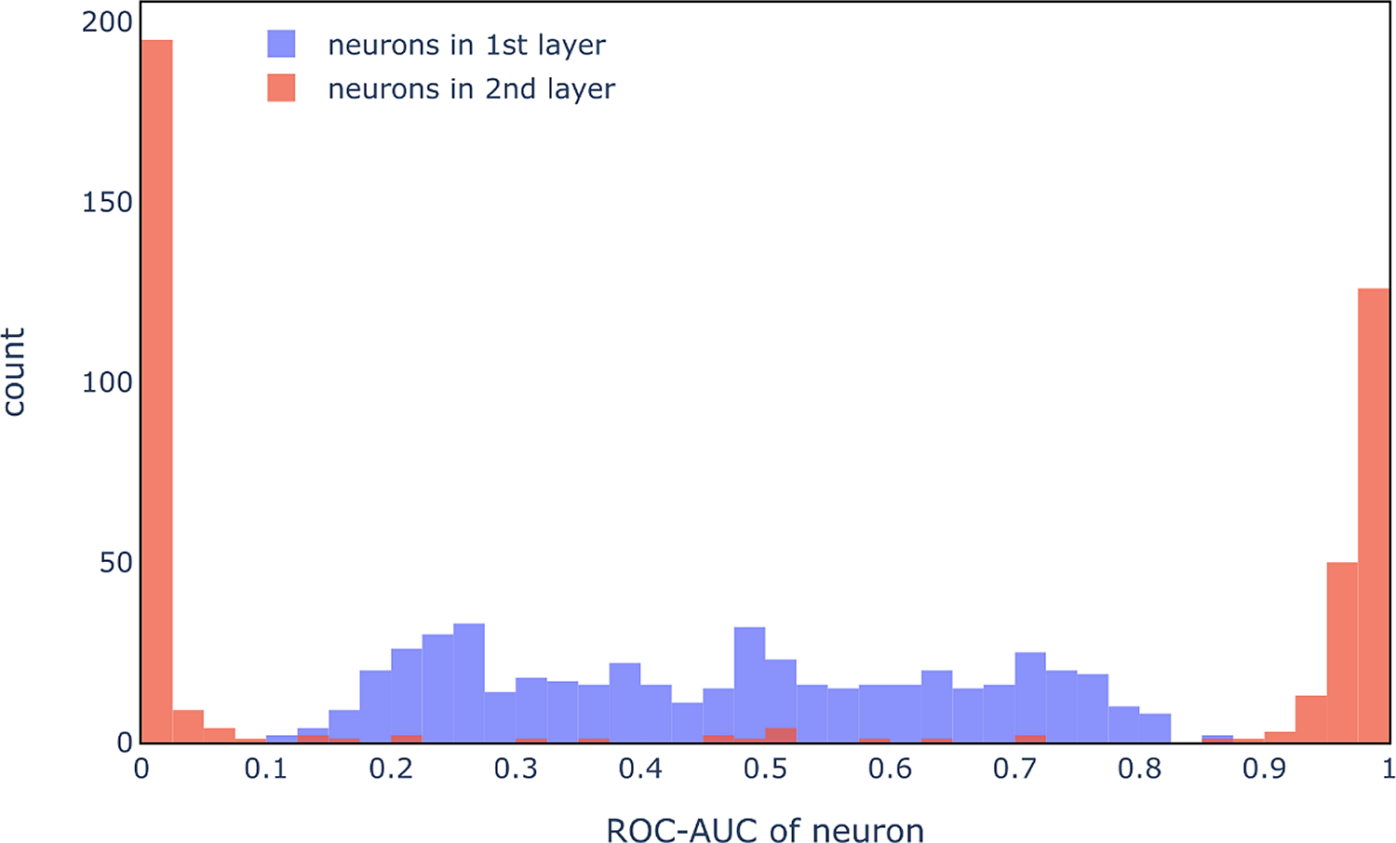
ROC_AUC scores for individual neurons. Each neuron in the first and second hidden layers is evaluated as a classifier.

ROC-AUC scores for individual neurons were then examined to investigate if hidden neurons detect specific chemical features. In this analysis, each hidden neuron is considered to be a classifier and the activation of the neuron for a compound is considered to be a prediction. An AUC score of 1 would mean that all toxic compounds cause a stronger activation than all nontoxic compounds (nonspecific detection of toxic compounds). On the other hand, an intermediate AUC (0.6–0.8) would mean that some but not all toxic compounds cause a strong activation which would be observed if only one (or a few) chemical features related to the toxicity are detected in the neuron. Equivalently, an AUC score of 0 would mean that all nontoxic compounds cause higher activation than toxic compounds. AUC scores for hidden neurons of the first and second hidden layers evaluated on training compounds are summarized in Figure 13.

It can clearly be seen that while neurons in the first hidden layer detect one or a few specific features for mutagenicity, neurons in the second hidden layer seem to directly detect mutagenicity (AUC close to 1) or absence of mutagenicity (AUC close to 0). It seems that in this model, neurons in the second hidden layer aggregate chemical features detected in the first layer instead of detecting novel chemical features. Similar observations were made on models trained on endpoints from the ChEMBL and ToxCast data sets (examples are shown in the Supporting Information: adenosine A1 receptor in Figure S8; ATG_ERa_TRANS_up in Figure S9).^60−62^ Hence, applying IG_hidden to the first layer can be expected to be sufficient to detect relevant chemical features, and no attempts were made to extract chemical substructures from neurons in the second hidden layer.

## Discussion and Conclusions

Although there has been significant interest in the use of NNs across a wide range of domains they are notorious as being “black boxes” with little or no rationale provided for the predictions made. As such, although they may provide more accurate predictions than traditional ML methods, they may not be the method of choice. In chemoinformatics applications, such as bioactivity or toxicity prediction, it can be more useful to understand the reasons why a certain prediction has been made as this may then inform future experiments, for example, in lead optimization, this information can be used to determine which parts of a molecule should be retained or changed as a project progresses. Furthermore, having the ability to interpret predictions is important in the regulatory context relating to the toxicity of chemicals.

Previous work aimed at opening the “black box” of NNs when applied to QSAR predictions has been based on assigning importance to the input features, such as the bits in a fingerprint, by integrating the weights over successive layers of the NN. While these can be used to assign atom attributions, they do not make direct use of the hidden layers of a NN where the input features are weighted, and combinations of features are identified as being associated with predictions. Here we have developed a method to exploit the information in hidden layers of a NN to assemble substructures from sets of highly weighted bits learned by individual neurons. The substructures are assembled by identifying co-occurrences of highly weighted bits in training compounds that strongly activate the neuron. The substructures are then associated with the hidden neurons of the NN and can be used to provide explanations for toxicity predictions when the NN is applied to previously unseen compounds. This approach was inspired by the method described in,^52^ and to our knowledge has not previously been attempted in the chemoinformatics field.

We have validated the method globally by comparing the set of substructures extracted from Ames toxicity training data that has been labeled according to Derek Nexus structural alerts which have been compiled manually. Substructures were found that are closely related to all but one of the expected 102 Derek Nexus alerts demonstrating that the data-driven approach is able to identify substructures that correspond to known toxicophores. We also evaluated the method locally by comparing the explanations provided for compounds not included in the training data and compared its performance against IG_input, a closely related and more established approach, which is based on the input features only. Both methods performed well in providing explanations for predictions that correspond closely to known toxicophores, measured using attribution ROC-AUC as the metric.^56^ However, neither of the methods was clearly superior, with each method performing better on certain mutagenicity alerts. Hence, it appears that leveraging information extracted from hidden neurons provides model explanations that are complementary to those found using input features only.

While IG_hidden yielded competitive performance to the established IG_input, we make several observations that make this approach challenging. First, it was observed that one neuron may be activated by a range of diverse chemical substructures related to the end point (i.e., different toxicophores). Hence, it is usually impossible to assign a single cause to the activation of a particular neuron. This is different from the input features where a Morgan fingerprint bit is assigned to a defined chemical environment (although bit collisions might occur). Moreover, relevant chemical features may activate a large number of different neurons across the NN. This means that many different neurons may be of relevance to understanding a given model prediction. A further observation is that it could be that none of the identified substructures associated with a highly activated neuron match the test compound. This is a limitation of the approach that could be mitigated by using more generic representations of substructures such as would be provided by SMARTS representations.^63^

When we extended the approach to investigate deep layers of NNs, it was observed that the individual neurons in the second hidden layer do not detect a subset of specific chemical features linked to the toxicity, but instead, they appear to aggregate all the chemical features found to be relevant for toxicity.

Benchmarking the performance of model interpretability techniques has been recognized as a crucial step to advance the state of the art.^64,65^ Here, we used an artificial data set (i.e., Derek Nexus alerts to determine class labels and ground truth atoms) which is related to a real toxicity end point (i.e., mutagenicity). This end point is interesting for benchmarking, as the model is required to learn a wide range of different chemical features. Although the data set used is proprietary, publicly available toxicity alerts (e.g., ToxAlerts, OECD QSAR Toolbox)^35,66^ may be leveraged as useful public benchmark data sets.

We further note that several challenges remain when attempting to evaluate the quality of model explanations. When a model explanation does not match the true cause of toxicity, this may be either because the model made the prediction for the wrong reason (see Clever Hans effect^67^) or because the explanation method does not correctly capture the model behavior. The inability of a model to correctly predict toxicity may be due to a limited number of training examples (e.g., for a certain chemical class) or to errors in the data (e.g., due to experimental variability). In our study, the quality of model explanations dropped slightly when moving from the Derek Nexus data set (where the labels correspond to well-defined chemical rules) to the experimental Ames data set (which is prone to experimental errors). Nevertheless, the model explanations were still in good agreement with the known toxicophores which suggests that the explanations for this data set were not strongly impacted by data errors.

Finally, the interpretation method has been developed to explain classification models. However, the IG technique has been successfully applied to regression models,^68^ and we believe that our method should hence also be applicable to regression models, although this was beyond the scope of this work.

To conclude, our study presents a novel method to extract learned chemical information from hidden layers of NNs and use these to explain model predictions. We believe that this paradigm can complement more established techniques for understanding NN models for toxicity prediction.

## Data Availability

The data set with experimental data as well as the trained neural network model are available. We further provide the code used to generate model explanations with both IG_input and IG_hidden in the accompanying GitHub repository (https://github.com/mowal/interpretability_learned_chem_feat). The labels from the proprietary Derek Nexus software and the models trained on this data are not available.

## Supporting Information Available

Exploration of most relevant training compounds and FP bits for further hidden neurons; evaluation of model explanations on the test set (Derek Nexus labels); evaluation of model explanations for the model trained on experimental Ames labels; evaluation of model explanations for the two-layer model (Derek Nexus labels); and evaluation of individual neurons as classifiers for further data sets (PDF)

## Notes

For the purpose of open access, the author has applied a Creative Commons Attribution (CC BY) license to any Author Accepted Manuscript version arising from this submission.

## Abbreviations

CAS: Chemical Abstracts Service
CGX: Carcinogenicity Genotoxicity eXperience
CNN: convolutional neural network
DNN: deep neural network
EURL-ECVAM: EU reference laboratory for alternatives to animal testing
FC: formal concept
FCA: formal concept analysis
FN: false negative
FP: fingerprint
FPR: false positive rate
ICH: International Council for Harmonisation of Technical Requirements for Pharmaceuticals for Human Use
IG: integrated gradients
InChI: International Chemical Identifier
ISSSTY: Istituto Superiore di Sanità *Salmonella typhimurium*
LIME: Local Interpretable Model-Agnostic Explanations
MCC: Matthews correlation coefficient
ML: machine learning
NN: neural network
OECD: Organization for Economic Co-operation and Development
ROC-AUC: area under the receiver operating characteristic curve
SDF: structure-data file
SHAP: Shapley Additive exPlanations
SMARTS: SMiles Arbitrary Target Specification
SMILES: Simplified Molecular-Input Line-Entry System
SOHN: Self-Organizing Hypothesis Network
TP: true positive
TPR: true positive rate
t-SNE: t-distributed stochastic neighbor embedding
