# Supplementary material for: Interpreting Neural Network Models for Toxicity Prediction by Extracting Learned Chemical Features
Source: J Chem Inf Model. 2024 Apr 30;64(9):3670–88. doi: 10.1021/acs.jcim.4c00127 (PMC11094726; doi:10.1021/acs.jcim.4c00127)
Supplement: Supplementary file 1 — ci4c00127_si_001.pdf [file ci4c00127_si_001.pdf]

Supporting Information

Interpreting Neural Network Models for Toxicity Prediction by Extracting Learned Chemical Features

*Moritz Walter[a], Samuel J. Webb[b], Valerie J. Gillet[a]**

[a] University of Sheffield, Information School, The Wave, 2 Whitham Road, Sheffield S10 2AH, UK

[b] Lhasa Limited, Granary Wharf House, 2 Canal Wharf, Leeds LS11 5PY, UK

*corresponding author: Valerie J. Gillet. v.gillet@sheffield.ac.uk

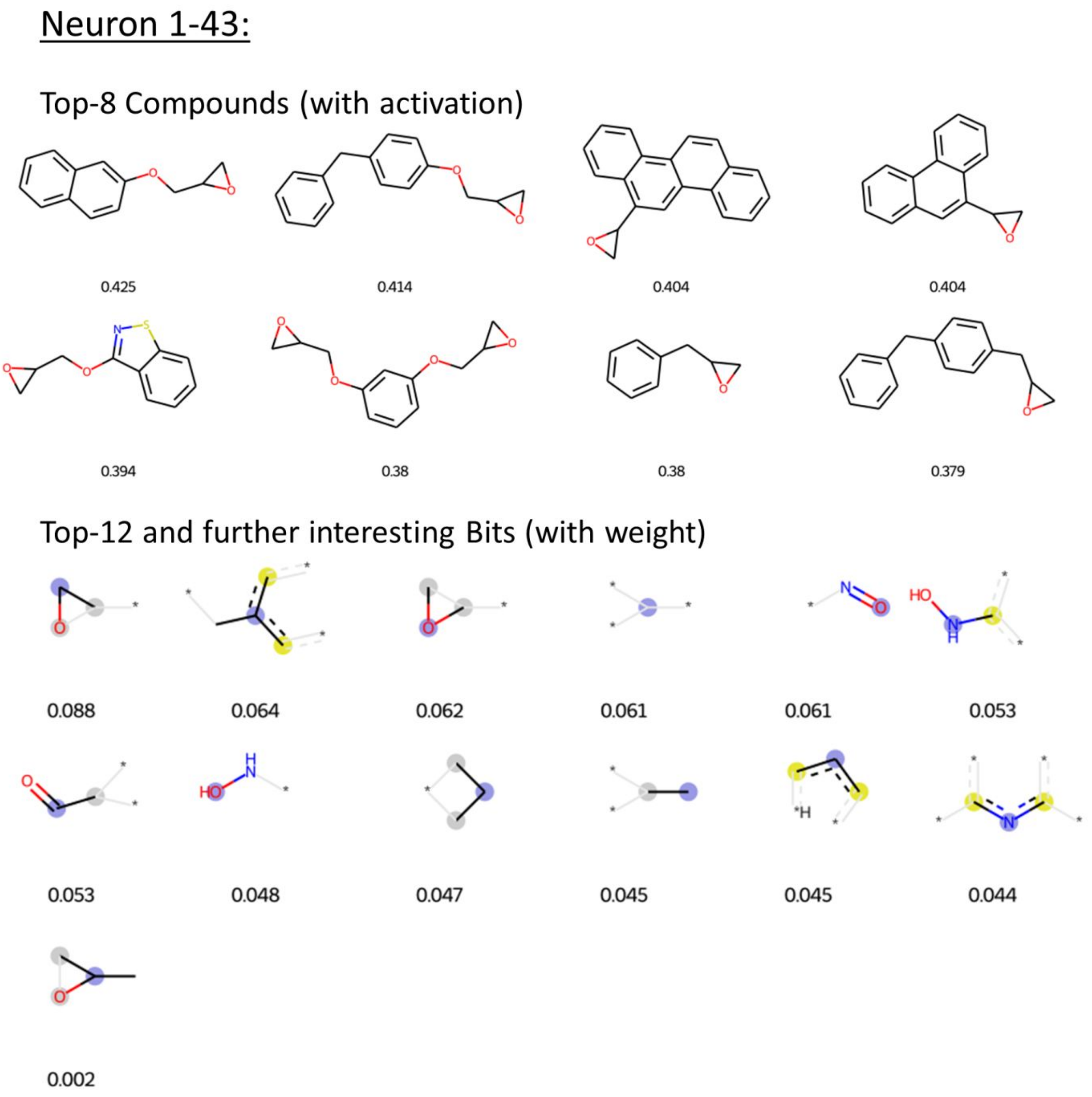

Figure S1 Most relevant training compounds and FP bits for neuron 1-143. Shown are the Top-8 training compounds (strongest activation) and the Top-12 FP bits (highest weights), as well as a further bit linked to the epoxide group (bottom row of lower panel).

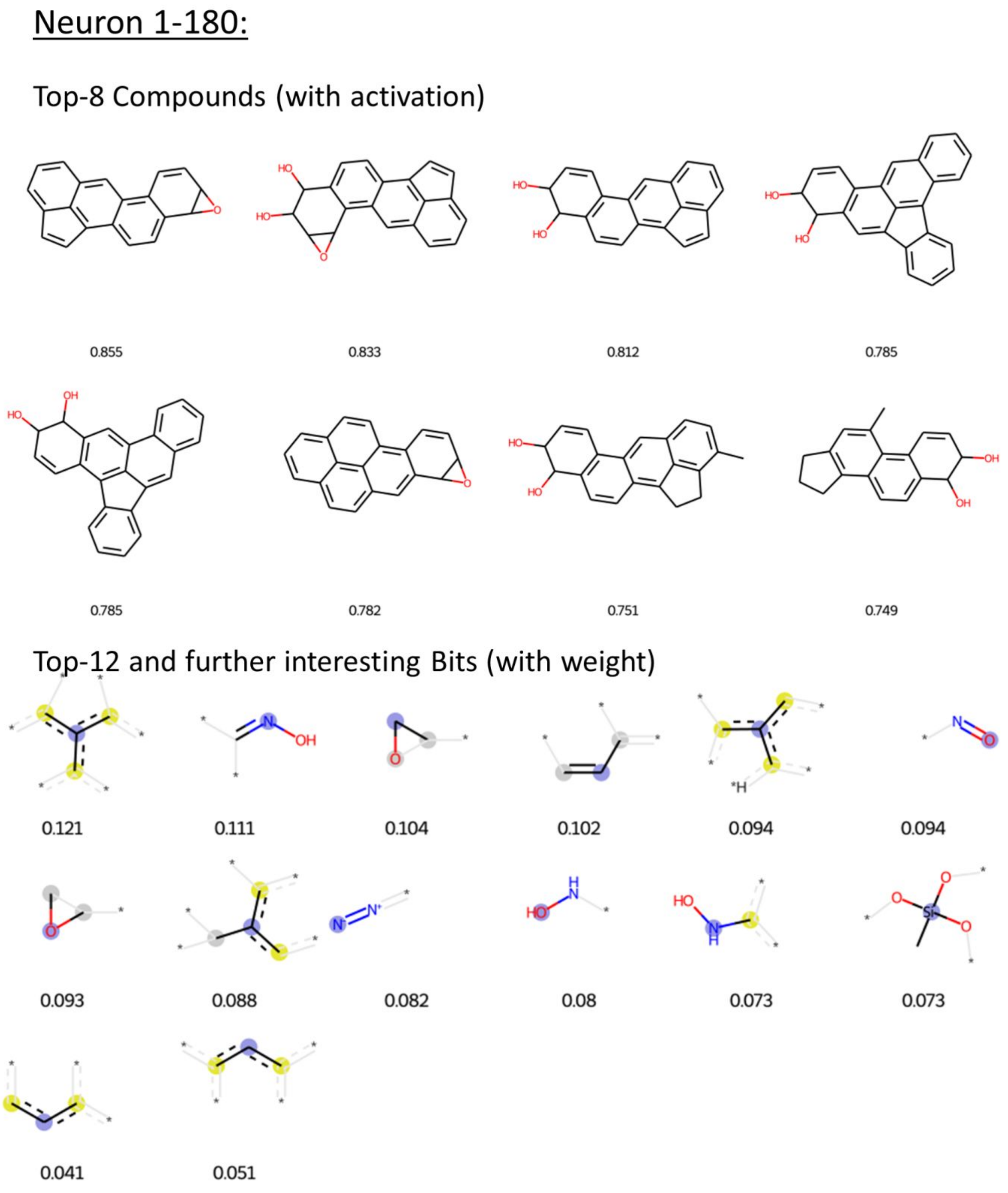

Figure S2 Most relevant training compounds and FP bits for neuron 1-180. Shown are the Top-8 training compounds (strongest activation) and the Top-12 FP bits (highest weights), as well as two further bits linked to polycyclic aromatic systems (bottom row of lower panel).

Neuron 1-69:

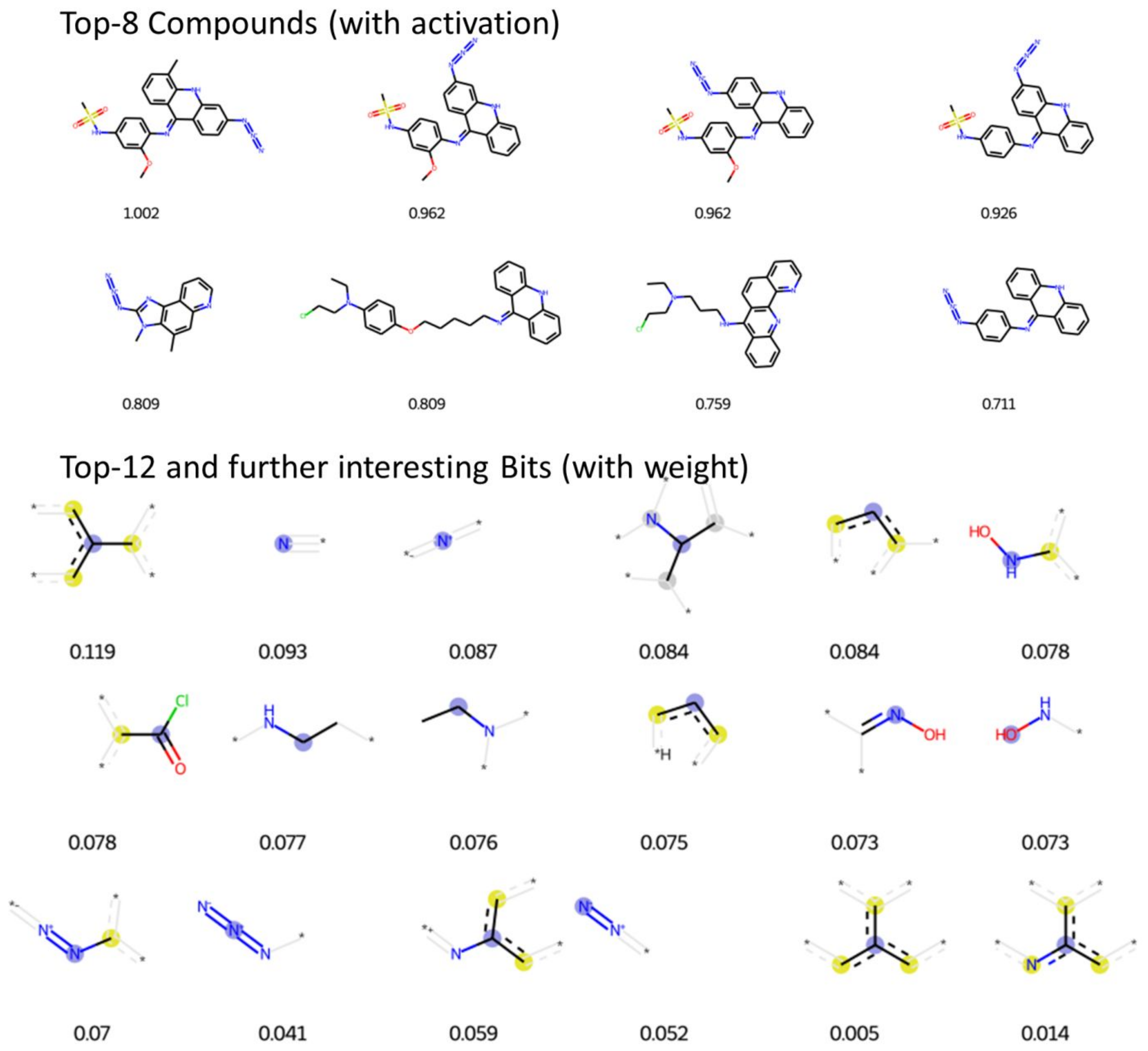

Figure S3 Most relevant training compounds and FP bits for neuron 1-69. Shown are the Top-8 training compounds (strongest activation) and the Top-12 FP bits (highest weights), as well as six further bits linked to the azide group or polycyclic aromatic systems (bottom row of lower panel).

Neuron 1-71:

Top-8 Compounds (with activation)

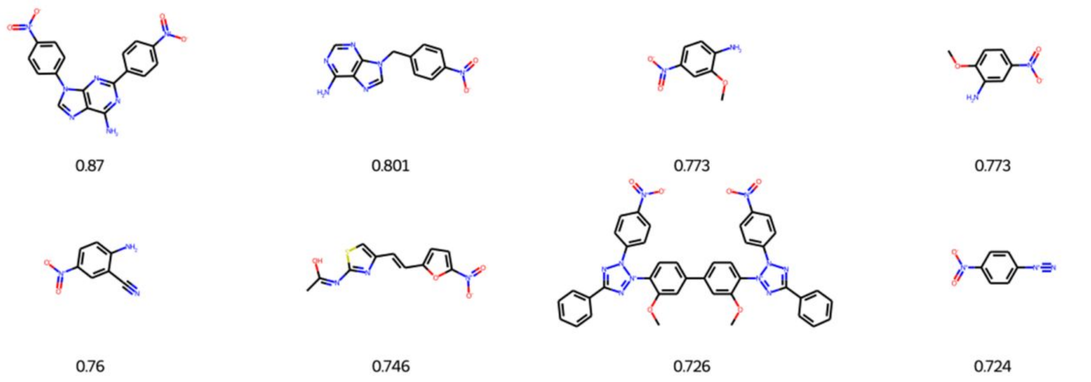

Top-12 and further interesting Bits (with weight)

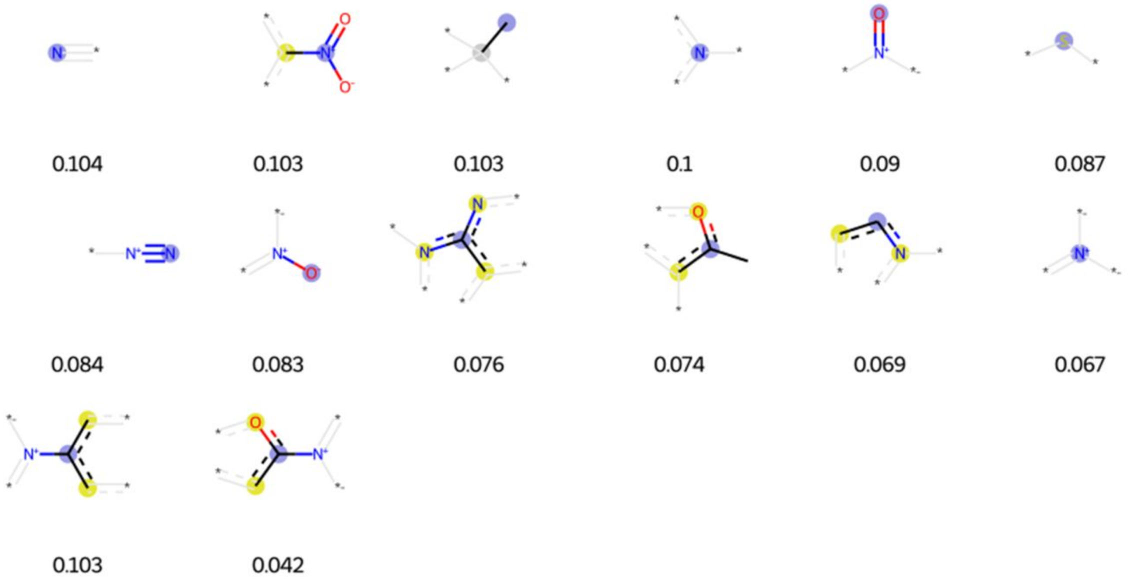

Figure S4 Most relevant training compounds and FP bits for neuron 1-71. Shown are the Top-8 training compounds (strongest activation) and the Top-12 FP bits (highest weights), as well as two further bits linked to the aromatic nitro group (bottom row of lower panel).

Table S1 Evaluation of model explanations on the test set.[a]

	Median AUC	AUC ≥ 0.8	Median alert AUC	Alert AUC ≥ 0.8
IG_input	**0.965**	**0.765**	0.838	**0.702**
IG_hidden	0.938	0.725	**0.852**	0.532

[a]Shown are the median attribution AUC across TP compounds, the proportion of compounds with an attribution AUC of ≥0.8, the median alert attribution AUC and the proportion of alerts with an attribution AUC≥0.8.

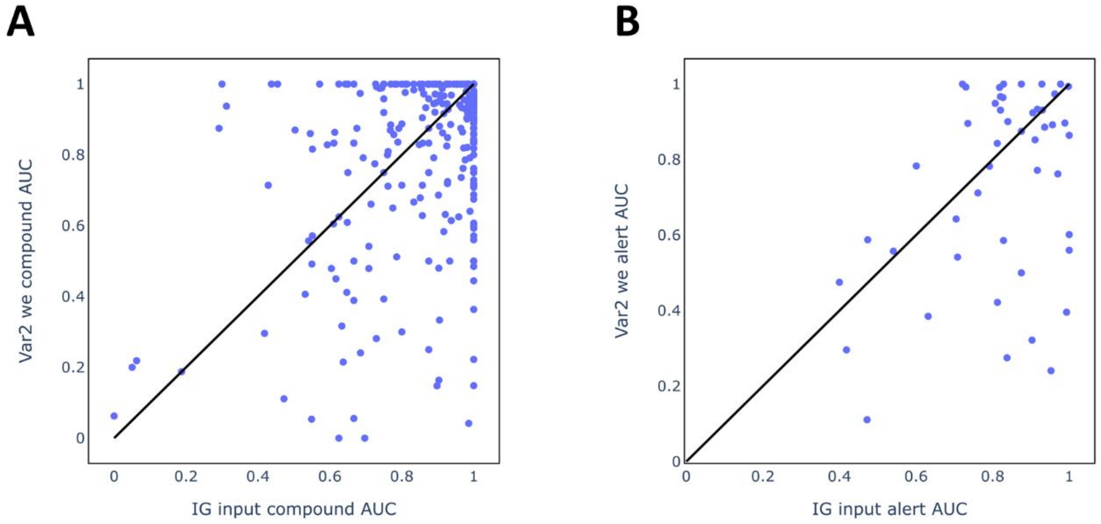

Figure S5 Attribution AUC scores of individual compounds (A) and alerts (B) evaluated on the test set.

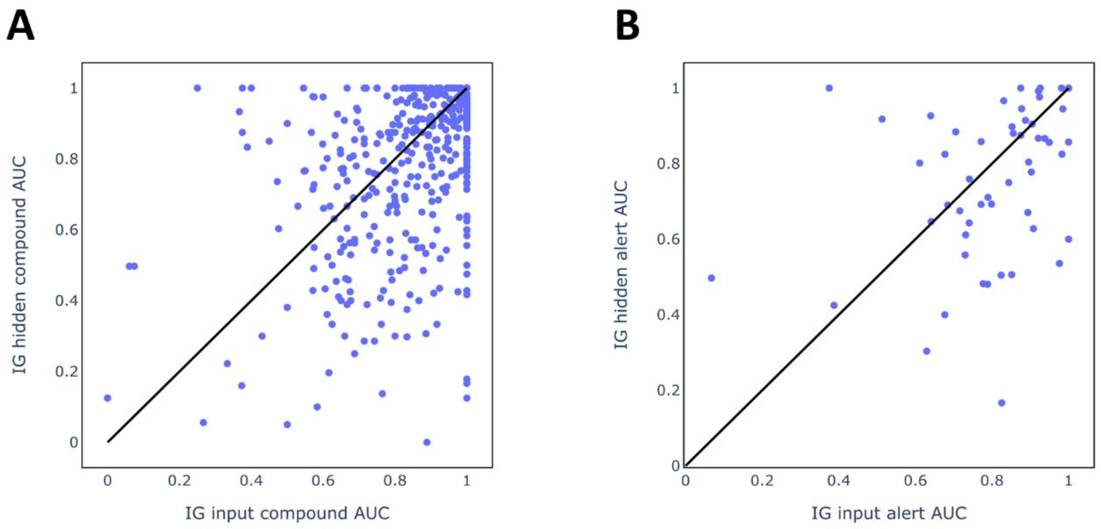

Figure S6 Attribution AUC scores of individual compounds (A) and alerts (B) for the model trained on experimental Ames labels.

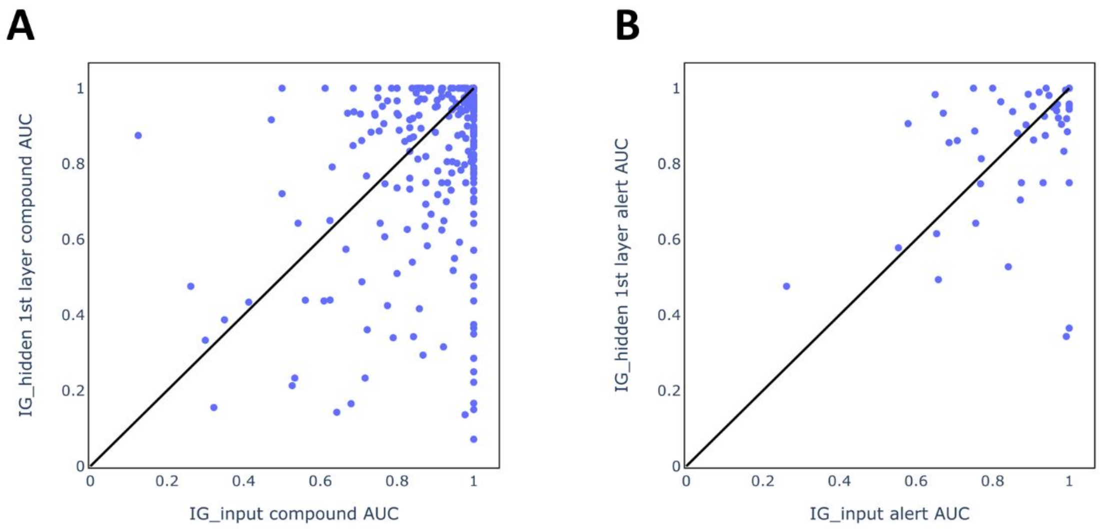

Figure S7 Attribution AUC scores of individual compounds (A) and alerts (B) for the 2-layer model trained on Derek Nexus labels.

Shown in Figure S8 and Figure S9 are ROC-AUC scores equivalent to those for the Derek Nexus label dataset shown in Figure 13. The data was taken from Reference 51 (https://doi.org/10.1186/s13321-018-0325-4) (Adenosine A1 receptor) and Reference 53 (https://doi.org/10.1039/c7sc02664a) (ATG_Era_TRANS_up, ToxCast data in the MoleculeNet benchmark data). The particular endpoints have been selected because at least 2000 labels with at least 10% actives were available. The datasets were randomly split into training (80%), validation (10%) and test (10%) and a 2-layer neural network was trained.

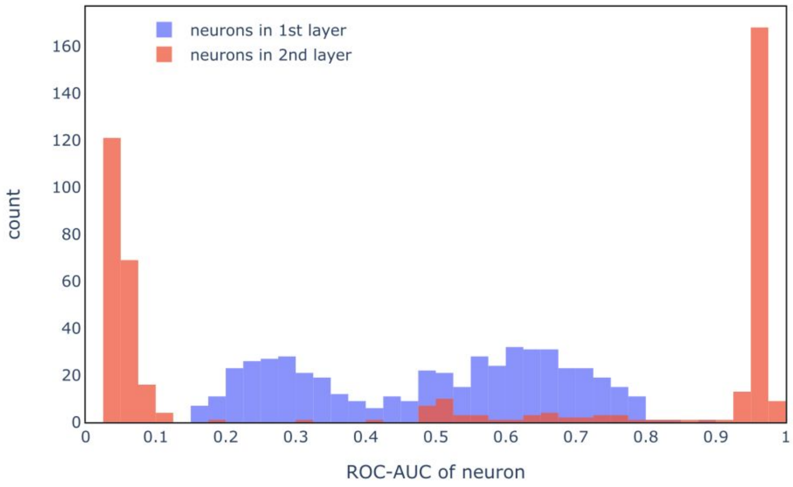

Figure S8 ROC_AUC scores for individual neurons: Adenosine A1 receptor data.

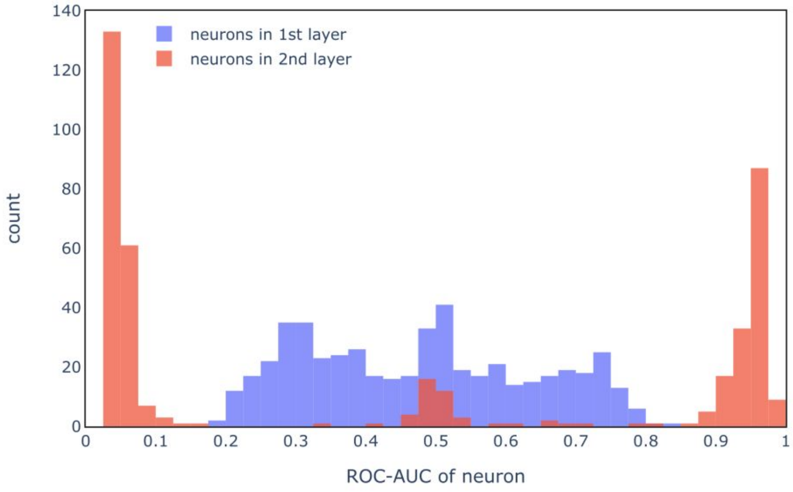

Figure S9 ROC_AUC scores for individual neurons: ATG_Era_TRANS_up.
